# Developing an evidence-based clinical pathway for the assessment, diagnosis and management of acute Charcot Neuro-Arthropathy: a systematic review

**DOI:** 10.1186/1757-1146-6-30

**Published:** 2013-07-30

**Authors:** Tamara E Milne, Joseph R Rogers, Ewan M Kinnear, Helen V Martin, Peter A Lazzarini, Thomas R Quinton, Frances M Boyle

**Affiliations:** 1 Podiatry Department, Ipswich General Hospital, Brisbane, Australia; 2 Podiatry Department, Launceston General Hospital, Launceston, Australia; 3 Podiatry Department, The Prince Charles Hospital, Brisbane, Australia; 4 Allied Health Research Collaborative, The Prince Charles Hospital, Brisbane, Australia; 5 School of Clinical Sciences, Queensland University of Technology, Brisbane, Australia; 6 Prosthetics, Orthotics and Podiatry Department, Princess Alexandra Hospital, Brisbane, Australia; 7 School of Population Health, University of Queensland, Brisbane, Australia

**Keywords:** Charcot Neuro-Arthropathy, Management, Clinical pathway, Diabetes

## Abstract

**Background:**

Charcot Neuro-Arthropathy (CN) is one of the more devastating complications of diabetes. To the best of the authors’ knowledge, it appears that no clinical tools based on a systematic review of existing literature have been developed to manage acute CN. Thus, the aim of this paper was to systematically review existing literature and develop an evidence-based clinical pathway for the assessment, diagnosis and management of acute CN in patients with diabetes.

**Methods:**

Electronic databases (Medline, PubMed, CINAHL, Embase and Cochrane Library), reference lists, and relevant key websites were systematically searched for literature discussing the assessment, diagnosis and/or management of acute CN published between 2002-2012. At least two independent investigators then quality rated and graded the evidence of each included paper. Consistent recommendations emanating from the included papers were then fashioned in a clinical pathway.

**Results:**

The systematic search identified 267 manuscripts, of which 117 (44%) met the inclusion criteria for this study. Most manuscripts discussing the assessment, diagnosis and/or management of acute CN constituted level IV (case series) or EO (expert opinion) evidence. The included literature was used to develop an evidence-based clinical pathway for the assessment, investigations, diagnosis and management of acute CN.

**Conclusions:**

This research has assisted in developing a comprehensive, evidence-based clinical pathway to promote consistent and optimal practice in the assessment, diagnosis and management of acute CN. The pathway aims to support health professionals in making early diagnosis and providing appropriate immediate management of acute CN, ultimately reducing its associated complications such as amputations and hospitalisations.

## Background

Charcot Neuro-Arthropathy (CN) is one of the more devastating complications affecting patients with diabetes and peripheral neuropathy [1]. It is a progressive, destructive condition that is characterised by acute fractures, dislocations and joint destruction in the weight-bearing neuropathic foot [2]. The acute phase is often misdiagnosed and can rapidly lead to severe foot deformity, ulceration and amputation [1,3,4]. Early diagnosis and management of acute CN is therefore imperative to avoid the rapid progression towards permanent foot deformation and its associated complications [5].

There are many reported aetiologies of CN, however in modern western societies diabetes mellitus has become the leading cause [1,5-7]. The true prevalence of CN is unknown, most likely due to a high incidence of mistaken or delayed initial diagnosis [7], but a number of population-based studies have reported an estimated prevalence of 0.4-13% in patients with diabetes [7,8].

To date, best practice assessment, diagnosis and management of acute CN appears to be influenced more by expert consensus than a rigorous evidence-base [1,5,6]. This may be because acute CN is considered one of the more rare complications of those caused by diabetes and thus tends to fall outside of the existing national guidelines or systematic reviews on diabetic foot complications [9]. This paper therefore aims to systematically review current relevant literature and develop an evidence-based clinical pathway for the assessment, diagnosis and management of acute CN in patients with diabetes.

## Methods

### Search strategy

A systematic review of the most relevant CN literature published between 2002-2012 was undertaken in the process of developing the clinical pathway. The search strategy was designed to identify relevant literature that focussed on the clinical assessment, diagnosis and/or conservative management of acute CN. For the purpose of this study the terms Charcot, Arthropathy, Neuroarthropathy, Osteoarthropathy, Neuro-Osteoarthropathy, and Neurogenic-Arthropathy were used interchangeably. The subsequent clinical pathway was guided by the recommendations specified by the National Health and Medical Research Council of Australia (NHMRC), 1999 [10,11].

Electronic databases (Medline, PubMed, CINAHL, Embase and Cochrane Library: Databases of Systematic Reviews) were searched for relevant literature by the first author in August 2012. Key search terms used were Charcot, Arthropathy, Neuroarthropathy, Osteoarthropathy, Neuro-Osteoarthropathy, and Neurogenic-Arthropathy. The search strategies for each database are summarised in Additional file 1. The exclusion criteria included papers published prior to 2002, not written in English, non-diabetes papers, or papers discussing the surgical management only of acute CN. As the focus of this paper was providing a contemporary clinical pathway for non-surgical health professionals, the authors considered the last decade of publications to be appropriate and to exclude surgical papers.

The initial search was intentionally broad in order to identify all literature pertaining to CN, and thus included both empirical evidence and expert opinion. To ensure completeness, the first author hand searched the reference lists of the initial manuscripts identified, searched web pages of relevant diabetes organisations for clinical practice guidelines, and contacted local and international experts in the field in an effort to identify any literature that may not have been identified in the initial search.

### Study selection

All titles and abstracts retrieved by the initial search were scanned by the first author using the following screening question: *Does the article discuss the clinical assessment, diagnosis and/or conservative management of acute CN in the diabetic foot?* If the article was deemed to meet the screening question, the first author retrieved the full text for quality assessment by the co-authors.

### Quality assessment

Co-authors, with expertise in diabetes related foot complications (TM, JR, EK, HM, PL, TQ), reviewed all identified full texts. At least two blinded co-authors independently reviewed each included article to assess its relevance and quality, and grade its level of evidence according to the NHMRC guidelines [9-11]. Table 1 provides definitions for the NHMRC levels of evidence [9,10]. Firstly, co-authors were required to review the full text to ensure it met all original inclusion criteria and to specifically exclude articles that focussed only on surgical management of CN or CN in non-diabetes populations. Secondly, co-authors were asked to assess if the article was of adequate quality or methodologically sound. In consideration of the small amount of literature published on CN, the definition of methodologically sound was broadened to exclude only articles not reporting methods or procedures (for example letters to the editor or commentaries). Lastly, the co-authors rated the article to assign it a level of evidence according to NHMRC guidelines [10,11]. Any inconsistencies between the assessments of manuscripts were resolved by the assessment of a third co-author.

**Table 1:** NHMRC levels of evidence

**Level of evidence**	**Definition**
I	A systematic review of level II studies
II	A randomised controlled trial
III	A pseudorandomised controlled trial (i.e. alternate allocation or some other method)
III-2	A comparative study with concurrent controls (i.e. non-randomised experimental trial, cohort study, case–control study)
III-3	A comparative study without concurrent controls (i.e. historical cohort study, two or more single arm study)
IV	Case series
EO	Expert opinion – where evidence was absent or unreliable and advice was formulated based on the clinical judgement and experience of experts in the field

### Data extraction

Literature that met the final inclusion criteria was then used to construct the clinical pathway. In an attempt to aid clinical management, the authors decided to base the development and flow of the pathway on the clinical phases evident in current general clinical management. These phases include assessments, investigations, diagnosis and management. Any common recommendations emanating from the final literature search were identified by the first and second authors and entered into the clinical domains. Clinical recommendations on the pathway were also welcomed from experts where quality evidence was lacking. The recommendations were prioritised according to level of evidence and relevance to the clinical pathway (Additional file 2, Additional file 3, Additional file 4). The final pathway was agreed to by the consensus of all co-authors.

## Results

A total of 267 manuscripts were identified from the initial search strategy. Of these, 117 (44%) were assessed to meet the final inclusion criteria and were used in the development of the clinical pathway. The 150 (56%) articles excluded were either considered lacking in quality or did not meet the final inclusion criteria. The large majority of manuscripts included were either expert opinion (67.5%) or level IV evidence (19%). Only three level II randomised control studies (RCT’s) were identified. Table 2 summarises the evidence levels of all included manuscripts. Table 3 summarises the country of publication origin of all included manuscripts. Most manuscripts were published in either the USA (50%) or UK (26%).

**Table 2:** Evidence levels of included manuscripts

**Level of evidence**	**Included manuscripts (117)**
I	0.0% (0)
II	2.5% (3)
III	11.0% (13)
IV	19.0% (22)
EO	67.5% (79)

**Table 3:** Country of publication of included manuscripts

**Country of publication**	**Included manuscripts (117)**
USA	50% (58)
UK	26% (31)
Germany	7% (8)
Netherlands	6% (7)
Israel	2% (2)
Scandinavia	2% (2)
Australia	1% (1)
Canada	1% (1)
China	1% (1)
France	1% (1)
Hong Kong	1% (1)
India	1% (1)
Morocco	1% (1)
Sweden	1% (1)
Switzerland	1% (1)

**Figure 1:**
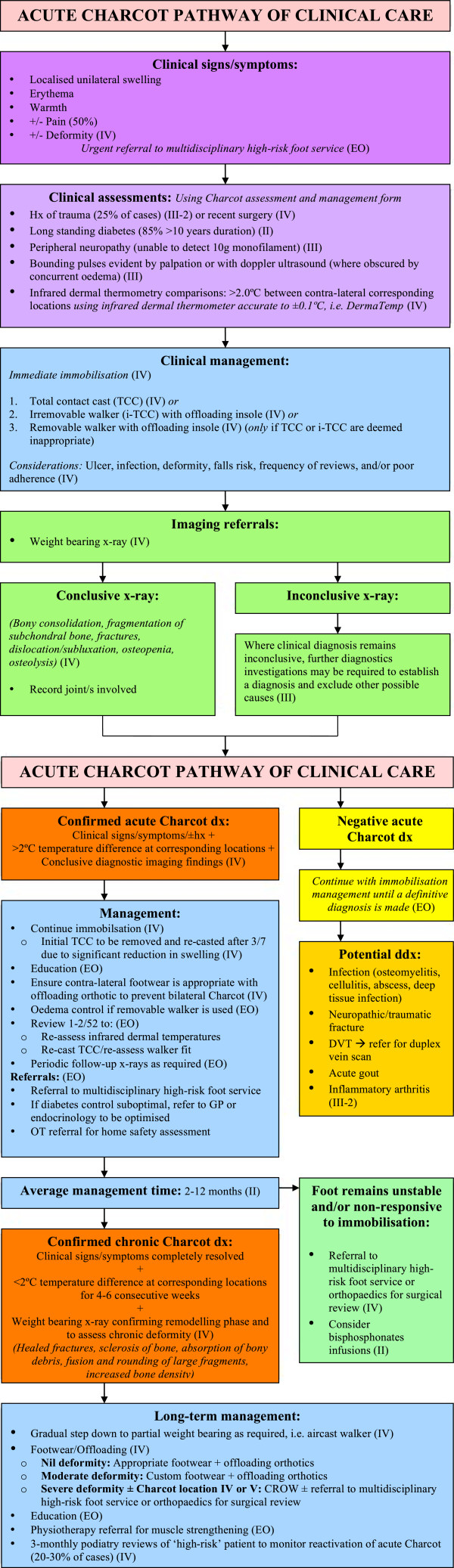
Acute Charcot Pathway of Clinical Care.

The pathway is divided into the four key phases for the clinical management of acute CN that have been addressed by the included literature. These phases are 1) Assessment, 2) Investigations, 3) Diagnosis, and 4) Management of acute CN. Unfortunately, some areas pertaining to these phases of management are devoid of quality research and in these instances low-level evidence, such as expert opinion, was utilised. The clinical pathway is presented in Figure 1.

### Assessment

#### Clinical signs & symptoms

##### Localised unilateral swelling, erythema, warmth, +/− pain (50%), +/− deformity: level of evidence = IV

It is well reported that acute CN characteristically presents with localised swelling, erythema and increased temperature (>2°C compared to the contralateral foot) to the affected foot [1,3,12,13]. Owing to the presence of peripheral neuropathy, pain may not always be present (reportedly in only 50% of cases) or will be less than expected given the severity of the clinical findings [12,14,15]. The diagnosis of acute CN is primarily dependant on this initial clinical presentation and therefore requires high clinical suspicion by the treating clinician for all patients with diabetes and peripheral neuropathy who present with these clinical signs and symptoms [14]. More advanced presentations of acute CN may also present with obvious foot deformity, including the characteristic ‘rocker-bottom’ deformity that is emblematic of CN [16].

##### Urgent referral to multidisciplinary high risk foot service: level of evidence = EO

If acute CN is suspected, an urgent referral to a multidisciplinary high-risk foot service or specialist clinic is recommended for appropriate multidisciplinary management of this complex condition [9,11,12,17,18].

#### Clinical assessments

##### History of trauma (25-50%): level of evidence = III-2 or recent surgery: level of evidence = IV

Preceding trauma may be recalled in as many as half of all cases of acute CN (25-50%) [3,15,16,19-22]. The role of trauma in an insensate extremity has been reported as an important factor in the pathogenesis of acute CN and should therefore be queried at the initial presentation [15,21]. However, due to the presence of an insensate extremity, it is important to consider recall bias as a cofounding factor and therefore a history of trauma may be unreliable [1]. In incidences where no trauma is recalled, repetitive micro-trauma on an insensate foot may be a contributing factor [9,21,22].

Recent foot surgery has also been described as a possible precipitating factor to acute CN [3,23]. The precise mechanisms by which surgery affects the pathogenesis of CN remain unclear, however it is reported that it may be associated with the local inflammation following surgery or alternatively as a result of the foot deformity following pedal amputation [1,24]. Pedal amputation can functionally compromise the foot leading to altered weight bearing forces that result in repetitive micro-trauma, a reported precipitating factor of acute CN [25,26].

##### Long-standing diabetes: level of evidence = II

The relationship between duration of diabetes and the onset of acute CN is well reported in a number of clinical trials and case series. Most commonly, at the time of onset patients with both Type 1 or Type 2 diabetes have been diagnosed for a period >10 years [8,13,27-30].

##### Peripheral neuropathy: level of evidence = III

The presence of peripheral sensory neuropathy is an important component for the onset of acute CN with no reported cases developing in its absence [1,8,31,32]. Peripheral sensory neuropathy can be accurately assessed using the Semmes-Weinstein 10 g monofilament [8,9,11,16,32].

##### Normal peripheral arterial perfusion: level of evidence-III

Generally, the acute CN foot has well preserved arterial perfusion. Pedal pulses can be palpated and are often described as ‘bounding’ in the acute CN foot, unless obscured by associated swelling. In this instance, the use of a doppler ultrasound may be required to assess arterial perfusion [1,8,9,11,31].

##### Infrared dermal thermometry comparisons >2°C: level of evidence = IV

Given the local inflammatory response during the acute phase of CN, temperature monitoring with the use of a handheld infrared dermal thermometre is a useful diagnostic assessment tool [33,34]. Infrared dermal thermometry comparisons between contralateral corresponding locations are typically >2.0°C in the affected foot [21,35,36]. Temperatures should be assessed approximately 15 minutes after the cast and footwear is removed and the use of an infrared dermal thermometer precise to ±0.1°C for a more accurate assessment is recommended [37]. Due to an absence of studies objectively determining or comparing different sites for temperature assessment, recommendations vary amongst the literature. Most frequently, however, skin temperatures are measured at the following 9 sites: dorsal mid foot, hallux, medial 1st metatarsal head, plantar 3rd metatarsal head, lateral 5th metatarsal head, 1st metatarsal-cuneiform joint, talonavicular joint, cuboid, plantar heel, and ankle [33,37].

#### Immediate clinical management

##### Immediate immobilisation: level of evidence = IV

In order to reduce the risk of severe chronic deformity, if acute CN is suspected, immediate immobilsation should be implemented until a definitive diagnosis is determined [12,38-40]. Immobilisation remains the cornerstone therapy for acute CN and is essential to break the cycle of repetitive trauma propagating the acute phase and to ultimately prevent the progression of deformity [1,41,42]. Options for immobilsation include the total contact cast and irremovable/removable walkers.

##### Total contact casts and irremovable walkers: level of evidence = IV

Total contact casts (TCCs) were originally referred to as the ‘gold standard’ immobilsation therapy for acute CN, due to their custom and irremovable nature [43-46]. The TCC is a custom moulded cast, commonly using plaster of Paris or fibreglass, which maintains contact with the entire planter service of the foot and lower limb [43-46]. The TCC immobilises the affected foot and ankle, reduces plantar foot pressures and swelling, protects from additional trauma, and maintains patient mobility [42,47].

An alternative to the TCC is the instant total contact cast (iTCC) which has been reported to be just as effective in immobilising the acute CN foot, as well as being more cost effective and requiring less skill to apply [1,48]. An iTCC consists of a prefabricated removable walker that is rendered irremovable by simply applying a layer of tape or fibreglass cast roll around the body of the walker to encourage patient compliance [2,46].

##### Removable walkers: level of evidence = IV

Prefabricated removable cast walkers have the benefit of immediate application without specialist skills and have been reported to be just as effective in offloading the diabetic foot, however patient adherence is often significantly reduced with these devices [31,41,47,48]. A large observational study of 288 patients with acute CN, reported that the use of irremovable offloading (TCC or iTCC) shortened the median time to resolution by approximately 3 months when compared with removable walkers [3]. This study highlights the issue with patient adherence when removable devices are prescribed. As a result, removable walkers should only be prescribed when TCCs or iTCCs are deemed inappropriate.

Previous studies have advocated complete non-weight bearing immobilsation with the use of crutches through the initial acute phase, however it has been reported that a three-point gait may in fact increase the load on the contralateral foot and thereby predispose the patient to bilateral acute CN [1,2,43]. Two recent case series demonstrated that ambulatory casting during the acute phase of CN does not negatively impact the outcome of CN and may in fact reduce the loss of muscle tone and bone density during immobilsation [1,42,44]. Given the paucity of empirical evidence regarding this issue, it is recommended that protective weight bearing should be advised at the discretion of the treating clinician.

##### Immobilisation considerations: level of evidence = IV

There are a number of important factors to consider before prescribing the most appropriate immobilsation device for the individual patient. The benefits of the TCC may be limited by the need for specially trained clinicians, available clinical time for application and product cost. In addition, cast changes are required within the first 3 days for the initial cast and 1-2 weekly thereafter to maintain proper fit and where necessary, permit wound management [1]. These frequent reviews may be particularly problematic for patients who live in rural or underserviced communities that are distant from specialised diabetic foot care clinics. In contrast, patients with current foot deformity may be at risk of secondary ulceration if fitted to a prefabricated walker and therefore a TCC may be the only appropriate means of immobilsation. Lastly, patients with CN often have increased instability and are at risk of falls as a result of multiple co-morbidities, including loss of proprioception and postural hypertension, and therefore aggressive cast immobilsation may not be appropriate and alterative modalities may need to be considered, such as a wheelchair [1,15,47].

### Investigations

#### Imaging referrals

##### Plain weight bearing radiographs: level of evidence = IV

If a patient presents with localised unilateral swelling, erythema and increased temperature in an insensate foot, plain radiographs are an important first line investigation and can be invaluable in ascertaining the presence of CN. In most cases, no further imaging studies are required to confirm diagnosis [1,2]. Characteristic radiographic signs of acute CN include bony consolidation, fragmentation of subchondral bone, fractures, dislocations, subluxations, osteopenia and osteolysis [35,49,50]. Although controversial, weight-bearing radiographs without immobilsation can be valuable in identifying subtle fractures, fragmentations and joint subluxation seen in very early stages of acute CN, which may not be present on standard non-weight bearing films. Additionally, joint deformity or collapse is often more accurately assessed in weight bearing views, and therefore weight-bearing views should be considered at the discretion of the clinician [21,39,44,50].

Where clinical diagnosis remains inconclusive at this time, further diagnostic investigations may be required to establish a diagnosis and exclude other possible causes.

##### Repeat radiographs at 2 weeks: level of evidence = EO

Normal radiographs at presentation does not necessarily exclude CN and it therefore may be important to perform further imaging investigations to confirm the diagnosis [15,49]. Easily available and inexpensive, repeat radiographs can be a valuable tool in confirming in the final diagnosis, especially in more remote locations where other diagnostic imaging modalities are not available. Repeat x-rays are generally obtained after 2 weeks of the initial investigation as the radiographic signs of acute CN generally become more conspicuous during this period [33,34].

##### Magnetic resonance imaging: level of evidence = III

Magnetic resonance imaging (MRI) represents a non-invasive and sensitive diagnostic tool in the study of bone marrow and soft tissue abnormalities, providing high quality images of the foot [51]. MRI has the ability to detect subtle changes in the early stages of acute CN, such as bone marrow oedema, before they are evident on plain radiographs [1,51,52]. This can play an important role in the early diagnosis of acute CN, when radiographs are inconclusive, thereby improving clinical outcomes [51,52]. MRI has also been reported to be both sensitive (77-100%) and specific (80%-100%) in the diagnosis of CN and osteomyelitis, a well reported challenge for most treating clinicians [1,11,49,53]. Where available and appropriate for use, MRI should be the imaging modality of choice for the diagnosis of acute CN when radiographs are inconclusive [1,9,11,33].

##### Nuclear medicine: level of evidence = IV

Nuclear medicine includes a number of diagnostic tests based on the use of radioisotopic tracers [1]. Nuclear medicine may play an important role in the diagnosis of acute CN where other imaging modalities, such as MRI, are contraindicated or unavailable [51]. Three-phase bone scans are highly sensitive (<100%) to acute bone pathology but lack specificity for acute CN [49,51]. For patients with low clinical suspicion of osteomyelitis and no signs of CN on initial radiographs, three-phase bone scans have proven to be a useful tool in assisting in the diagnosis. Alternatively, leukocyte-labelled bone or marrow scans (99 m Tc HMPAO or 111 Indium) provide improved specificity (69-80%) for distinguishing infection from acute CN and are a more appropriate imaging tool when underlying infection is suspected [1,8,18,49].

##### FDG-PET: level of evidence = IV

More recently, 18 F-fluorodeoxyglucose positron emission tomography (FDG-PET) has been recognised as having potential for differentiating acute CN from osteomyelitis [1,49]. A few recent studies have reported that combined FDG-PET may have several advantages over existing imaging techniques for diagnosing osteomyelitis and acute CN, including improved sensitivity (100%) and specificity (93.8%) for acute CN, high-quality images with detailed anatomic localisation, and fast results within 1.5 to 2 hours after the initiation of the examination [54-56]. However, the 2011 international task force consensus document on the Charcot foot in diabetes recommends that FDG-PET for the potential diagnosis of acute CN and osteomyelitis remains investigational at this time [1].

##### Bone biopsy: level of evidence = EO

Bone biopsy remains the only diagnostic method for definitive discrimination between osteomyelitis and CN. This is not, however, always appropriate and can potentially result in a number of secondary complications including infection, excessive bleeding, pain, fracture, or new onset of acute CN [53,57,58]. With these limitations in mind, bone biopsy should only performed if the diagnosis remains inconclusive after imaging is exhausted, or if osteomyelitis is likely [53].

#### Serology referrals

There is currently no universally accepted serology criterion for the diagnosis of CN; however, some studies have reported that serology markers may assist in narrowing the diagnosis [8,11,14,15,35,39,59].

##### Leukocytosis, C-reactive protein, and erythrocyte sedimentation rate: level of evidence = IV

Leukocytosis (WCC), an elevated C-reactive protein (CRP) and erythrocyte sedimentation rate (ESR), and recent unexplained hyperglycemia are all systemic responses to infection [11,14,39]. The utility of these inflammatory parameters for identifying infection has been supported throughout the literature and could therefore be a valuable assessment tool for differentiating between acute CN and infection [8,15,35,59]. Recent studies have demonstrated that there is dissociation between the local and systemic inflammatory response in acute CN, with serum WCC, CRP, and ESR values remaining within the reference range for patients with acute CN despite the presence of local inflammation [35,59]. Therefore, in the absence of elevated systematic inflammatory markers, infection may be an unlikely diagnosis and acute CN should be considered [21].

##### Glycosylated haemoglobin: level of evidence = IV

Chronic hyperglycaemia is a major incipient factor in the development of CN, a theory well supported by the literature [60]. One recent case study, reported that an elevated glycosylated haemoglobin (HbA1c) is associated with more than a 30% increase in the risk for developing CN [61]. As mentioned earlier, recent unexplained hyperglycaemia may also assist in the diagnosis of infection [14]. Clinicians have long recognised the importance of tight glycaemic control in reducing the risk of diabetic foot complications [61]. Therefore, as part of a multidisciplinary approach to the management of a patient with diabetes and suspected CN, it is recommended the HbA1c be assessed and, where necessary, optimised [2].

##### Electrolytes and renal function: level of evidence = IV

Diabetic nephropathy has been reported to be associated with an increased incidence of acute CN [28]. One case study reported that renal failure nearly doubled the risk of CN (OR 2.1, p < 0.001), suggesting that patients with co-existing renal failure be carefully monitored for signs of acute CN [61].

##### Calcium and vitamin D: level of evidence = EO

Pathological or traumatic fractures are a reported misdiagnosis of acute CN, often associated with deficiencies in calcium and vitamin D resulting in inadequate mineralisation of the bone [1,59,62]. Furthermore, if bisphosphonates are administered as part of the management plan, they require adequate levels of calcium and vitamin D to work effectively [6,63,64]. Therefore, investigating calcium and vitamin D levels may be beneficial in assisting with the diagnosis and/or directing the management plan.

##### Uric acid: level of evidence = EO

An acute gout attack may also masquerade as acute CN, however can be excluded by measurement of serum uric acid, which is typically raised in the presence of gout [15,39,65].

### Diagnosis

#### Acute CN diagnosis criteria

##### Level of evidence = IV

In the absence of rigorous evidence, the most commonly accepted criteria by treating clinicians for the diagnosis of acute CN is: a warm, swollen, erythematic foot (clinical signs), with or without any significant history of trauma or surgery, a temperature difference from the contralateral foot of > 2°C, and conclusive diagnostic images suggestive of acute CN [1,13,29,55]. In the presence of a wound or history of osteomyelitis, clinical suspicion and assessment of osteomyelitis should be considered [2,11,12,16].

#### Negative diagnosis

##### Level of evidence = EO

In the event that CN may not be the most likely diagnosis, experts in the field recommend continuing with immobilisation until a definitive diagnosis is made so that the risk of foot deformity or other associated complications can be avoided if in fact CN is later diagnosed [4,38].

#### Differential diagnosis

##### Level of evidence = III-2

Historically, misdiagnoses for acute CN have included infection (osteomyelitis, cellulitis, abscess, deep tissue infection), DVT, acute gout, neuropathic/traumatic fractures, sprain, or inflammatory arthritis [4,17,21,22,59]. One retrospective case series reported that 80% of patients with acute CN were initially misdiagnosed as having sprains (n = 11), DVT (n = 3), osteomyelitis (n = 4), tumour (3), cellulitis (n = 6), or rheumatoid arthritis (n = 2) [17]. Given its rare presentation, it is not surprising that a large number of cases of acute CN are initially misdiagnosed; however, this only further emphasises the need for high clinical suspicion when a patient with diabetes and neuropathy presents with the clinical signs and symptoms suggestive of acute CN.

### Management

#### Acute management

##### Continue immobilisation: level of evidence = IV

Immobilsation of the affected foot continues until complete resolution of the acute phase [3,31,42]. The cast is initially replaced (TCC) or re-fit (iTCC, removable walker) after the first 3 days due to the significant oedema reduction seen after this period. The cast is then replaced 1-2 weekly after this time, again to adjust for limb volume changes from oedema and to assess for any complications secondary to immobilsation [1,43].

##### Education: level of evidence = EO

Patient education regarding the diagnosis, estimated length of treatment and expected outcomes is an important component of CN management. If the patient understands the nature of this limb-threatening condition, they may be more motivated to adhere to the management plan. Emphasis on the importance of strict immobilisation, attending regular follow-up reviews and optimising glucose control may improve the outcome of CN [11,39,58,66].

##### Appropriate contralateral footwear: level of evidence = IV

Bilateral CN is reported in as many as 30% of cases [8,45,67]. As stated earlier, immobilsation therapy, especially with the use of crutches, has been reported to potentially increase the load on the contralateral foot and thereby predispose the patient to bilateral acute CN [1,2]. For this reason, prophylactic support with appropriate footwear and accommodative insoles is recommended for the contralateral foot to minimise the risk of bilateral acute CN [9,39,68,69].

##### Oedema control: level of evidence = EO

When a TCC is applied to immobilise the acute CN, the compression of the cast will assist in reducing the oedema present in the acute phase. However, when a prefabricated walker is used it is recommended that oedema be managed with alternative compression therapies such as elastic bandaging [31,70].

##### Regular reviews: level of evidence = IV

During the immobilisation period, regular reviews by a high-risk foot service are important to monitor the activity of the acute phase, review the management plan, and assess and manage any secondary complications [1]. Measuring skin temperature differences between the affected and the non-affected foot using an infrared dermal thermometre is an objective measure for monitoring reduction in inflammation during the acute phase of CN [21,34,66]. The literature suggests that elevated temperatures will correlate with the location of CN and that temperatures in the affected foot will decrease as acute CN progresses into the chronic phase [33]. TCC’s should be re-casted and the fit of walkers re-assessed at 1-2 weekly reviews to adjust to limb volume changes as the oedema subsides during immobilisation [2,43,44,71].

##### Periodic follow-up radiographs: level of evidence = EO

Following the initial diagnosis, follow-up radiographs of the affected foot every 4-6 weeks will monitor the progression of CN, as well as any changes in the architectural alignment and configuration of the foot [8,33,34,42,70]. However, given the paucity of empirical evidence recommending the benefit of periodic follow-up radiographs, these are performed at the discretion of the treating clinician.

##### Appropriate referrals: level of evidence = EO

Given the complexity of CN, a multidisciplinary approach to the holistic management of the patient is recommended [72]. Where appropriate, the authors recommend referrals to a multidisciplinary high-risk foot clinic [12,17,39], local general practitioner or specialist physician to optimise diabetes management and/or other relevant comorbidities [61,64,66,73], and occupational therapy for a home environment assessment, especially when crutches or a wheel chair are prescribed [15].

##### Bisphosphonates: level of evidence = II

There are currently conflicting reports on the clinical benefits of bisphosphonates for the management of acute CN [6]. Systematic reviews of clinical trials have indicated that bisphosphonates are ineffective and may even be harmful to the resolution time of the acute phase of CN [3,6,13]. In contrast, other studies of the same level of evidence have supported their use, suggesting that bisphosphonates may improve the resolution time of the acute phase by reducing skin temperature and disease activity [72,74-76]. Therefore, given the inconclusive evidence on their use, it is recommended that bisphosphonates be used at the discretion of the treating physician for cases of acute CN that are non-responsive to conservative immobilsation management.

##### Average management time: level of evidence = II

A number of clinical trials and case series have reported average management times for the complete resolution of CN between 2-12 months, with a period of 6 months being most commonly reported [3,13,17,27,28,31,42,47]. The literature suggests that the management time may be influenced by the location of CN, type of immobilsation used, and the stage of CN when immobilisation is implemented [3,17,20,39,47].

#### Chronic CN diagnosis criteria

##### Level of evidence = IV

The duration of immobilisation is guided by the clinical assessment that the acute phase has completely resolved [1]. This is evident by the resolution of all clinical signs and symptoms, stabilised contralateral skin temperatures, and evidence of healing on radiographs [13,31]. Previous studies have recommended a skin temperature difference between contralateral locations of <2°C for 2-4 consecutive weeks before transitioning patients from cast immobilisation to a removable walker or appropriate footwear [21,31-33]. Radiographs are an important tool in assisting in the diagnosis of chronic CN and are recommended once all clinical signs and symptoms have resolved [49]. Radiographic evidence of chronic CN includes healed fractures, sclerosis of bone, absorption of bony debris, fusion and rounding of large fragments, and increased bone density [15,49,68,76]. Feet with severe CN deformity are siginificantly associated with midfoot ulceration. Therefore, weight bearing radiographs of chronic CN may be more beneficial at this time to assess the presence and degree of deformity so that appropriate long term offloading can be prescribed [77].

#### Long-term management

##### Partial weight bearing: level of evidence = IV

Once the foot is stable, transition to protected weight bearing is generally advised before the patient steps down to footwear [2,30,66]. Aircast walkers or other similar prefabricated removable walkers have gained acceptance as useful protective modalities for this initial period of weight bearing [15,44,66]. Partial weight bearing has been reported to minimise the risk of reactivation of the acute phase if immobilsation is ceased too early [20,31].

##### Footwear and offloading: level of evidence = IV

Footwear is an important component of the long-term management of the insensate chronic CN foot, ensuring that it remains accommodated, offloaded and protected.

In patients with nil to minor foot deformity after the resolution of acute CN, prefabricated footwear with extra depth and a stiff rocker bottom walking sole may suffice. These shoes, when fitted with custom-molded, full-contact insoles, will adequately minimise load bearing and mobility of the foot during walking [2,8,20,30,44,47].

In the presence of moderate deformity, custom-made or modified shoes are generally necessary to accommodate the chronic foot deformity. Again, these shoes should be fitted with custom-molded, full contact insoles to minimise load bearing and mobility during walking [8,20,78].

Chronic CN that has resulted in severe foot deformities and/or CN that is located in the ankle or rear foot (location IV or V) can often be difficult to stabilise with footwear and typically requires more aggressive long term management such as a Charcot Restraint Orthotic Walker (CROW) to achieve stability and reduce the risk of reactivating the acute phase [1,15,40,46]. The CROW has proven to be useful in maintaining foot and ankle alignment in the instable or surgical corrected CN foot [78], however, where aggressive conservative management has failed, surgical correction of the deformity should be considered [1].

##### Education: level of evidence = EO

Patient education should form an essential component of the long-term management of these patients, focusing on the importance of appropriate footwear and offloading, regular follow up reviews, and the risk of further complications [11,12,66].

##### Rehabilitation: level of evidence = EO

Following an extended period of immobilsation, there will likely be wasting of the calf muscles, loss of bone density and joint stiffness [42,44]. Protective rehabilitation with a physiotherapist is recommended following the transition phase out of immobilisation, being cautious, however, of the risk of reactivation of the acute phase or ulceration of bony deformity by excessive rapid mobilisation during the early stages of rehabilitation [15,37].

##### Long term follow up and/or reactivation: level of evidence = IV

Three monthly podiatry reviews of these high-risk patients is advised to monitor for signs of recurrent or new episodes of CN, as well as any other diabetic foot complications [1,9,12,15]. Recurrence is reported in 15-30% of patients with a previous history of CN [3,15,69,79].

##### Surgical: level of evidence = IV

Typically, if the correct diagnosis is made in the acute phase of CN and conservative treatment is successful, surgery may be avoided and the risk of subsequent ulcerations and/or amputation may be decreased [30]. Surgical management is usually only considered in the chronic phase of CN where joint instability and/or severe deformity have failed to be effectively managed with a conservative approach [1,2,30]. Up to 50% of patients have been reported to undergo surgical procedures for long-term management of CN deformities and instabilities, most commonly occurring 4 years after the initial acute phase [2,30]. Surgery is generally avoided during the acute phase of CN due to the risk of mechanical failure or secondary infection [1].

## Discussion

Our systematic search of relevant literature highlights that CN continues to be a poorly understood disorder of the diabetic foot. Although recent clinical research has improved our level of knowledge regarding its etiology and management, there are still only a few high-level evidence-based studies regarding the assessment, diagnosis and management of acute CN [39]. As hypothesised, most literature pertaining to this field constitutes level IV or EO evidence and no systematic reviews were identified. Thus, this review begins to fill a gap commonly found in Australian and international diabetic foot complication guidelines that overlook the systematic review of CN [9].

CN continues to be a persistent challenge for clinicians, especially in its acute phase [6]. The literature reports that the diagnosis of CN is missed in as many as 79% of cases and an accurate diagnosis can be delayed up to 29 weeks. This highlights a clear gap in professional education, which this pathway hopes to address [4,21]. Moreover, it is well reported that patients with CN experience increased morbidity and mortality, a higher risk of amputations, and a reduced quality of life [5,6,30].

Currently, most available clinical guidelines on the management of acute CN are without a rigorous evidence-base, as displayed in the current pathway [2,18,80]. Therefore in the era of evidence-based medicine, this research has assisted in developing a comprehensive, evidence-based clinical pathway designed to promote consistent and optimal practice in the assessment, diagnosis and management of acute CN. However, it should be noted that whilst the pathway is there to assist clinician evidence based decision-making, clinical discretion is still very much required especially with the low level of evidence that most recommendations in this pathway carry.

A number of strengths and limitations of the review need to be acknowledged. Our review was deliberately broad and, given the paucity of methodologically rigorous studies in the field, included a review of expert opinion in order to provide a comprehensive basis for development of the pathway. All identified manuscripts were reviewed for relevance and quality by at least two members of the expert panel of podiatrists. However, this was done without the use of a formal quality appraisal tool and therefore the process was not validated. High-level evidence was prioritised in the development of the pathway, however, where evidence was lacking, expert opinion was sometimes the only option. In this case, it should be acknowledged that the recommendation is based on expert opinion rather than scientific evidence and this should be reviewed as new evidence becomes available [9]. Only studies published in English between 2002-2012 were included and it is therefore possible that some relevant research was excluded. However, hand searching of reference lists, exploration of grey literature and websites, and consultation with local and international researchers is likely to have minimised this possibility.

The authors recommended that the clinical pathway be now tested for validity & reliability, and used in larger longitudinal studies to investigate its impact on the devastating clinical outcomes of acute CN.

## Conclusions

CN appears to be a significantly under-recognised and under-researched complication of diabetes. Whilst CN remains a rare complication of diabetes, it results in significant levels of morbidity and mortality in the population of people with diabetes. Thus, immediate best practice management of this devastating complication is vital to improve clinical outcomes and patient quality of life. This systematic review, and subsequent pathway development, appears to be one of the first in the area of CN management. The pathway aims to support health professionals in making early diagnosis and providing appropriate immediate management of acute CN, ultimately preventing and reducing its associated complications such as amputations and hospitalisations. It is recommended that the pathway’s clinical outcomes are implemented and further researched to determine its applicability to minimise the devastating effects of CN.

## Abbreviations

CN: Charcot Neuro-Arthropathy; RCT: Randomised control trial; NHMRC: National health and medical research council; TCC: Total contact cast; iTCC: Instant total contact cast; MRI: Magnetic Resonance Imaging; FDG-PET: 18 F-Fluorodeoxyglucose positron emission tomography; WCC: Leukocytosis; CRP: C-Reactive protein; ESR: Erythrocyte sedimentation rate; HbA1c: Glycosylated haemoglobin; DVT: Deep vein thrombosis; CROW: Charcot Restraint Orthotic Walker.

## Competing interests

The authors have no relevant conflict of interest to disclose.

## Authors’ contributions

TM conceived, designed, performed literature search, quality assessments, contributed to discussion, wrote and reviewed/edited the manuscript. JR conceived, designed, performed quality assessments, contributed to the discussion, and reviewed/edited the manuscript. EK, HM, PL, TQ performed quality assessments, contributed to discussion and reviewed/edited the manuscript. FB designed, contributed to discussion and reviewed/edited the manuscript. All authors have read and approved the final manuscript.

## Supplementary Material

Additional file 1 Search strategies. Click here for file

Additional file 2 Level II evidence. Click here for file

Additional file 3 Level III-IV evidence. Click here for file

Additional file 4 Level EO evidence. Click here for file
