# Supplementary material for: Developing an evidence-based clinical pathway for the assessment, diagnosis and management of acute Charcot Neuro-Arthropathy: a systematic review
Source: J Foot Ankle Res. 2013 Jul 30;6:30. doi: 10.1186/1757-1146-6-30 (PMC3737070; doi:10.1186/1757-1146-6-30)
Supplement: Additional file 1 — Search strategies. [file 1757-1146-6-30-S1.doc]

**Additional file 1 - Search strategies**Medline:

1. ‘Charcot’
2. ‘Arthropathy’ or ‘Neuroarthropathy’ or ‘Osteoarthropathy’ or ‘Neuro-Osetoarthropathy’ or ‘Neurogenic Arthropathy’
3. Limit to English language and year 2002-‘current’
4. 1 and 2 and 3

PubMed:

1. Charcot AND (Arthropathy OR Neuroarthropathy OR Osteoarthropathy OR Neuro-Osetoarthropathy OR Neurogenic Arthropathy)

2. Limit to English language and year 2002-‘current’

CINAHL:

1. ‘Charcot’
2. ‘Arthropathy’ or ‘Neuroarthropathy’ or ‘Osteoarthropathy’ or ‘Neuro-Osetoarthropathy’ or ‘Neurogenic Arthropathy’
3. Limit to English language and year 2002-‘current’
4. 1 and 2 and 3

Embase:

1. ‘Charcot’ and ‘Neuropathic Joint Disease’
2. ‘Arthropathy’ or ‘Neuroarthropathy’ or ‘Osteoarthropathy’ or ‘Neuro-Osetoarthropathy’ or ‘Neurogenic Arthropathy’
3. Limit to English language and year 2002-‘current’
4. 1 and 2 and 3

Cochrane Library:

1. ‘Charcot’
2. Limit to English language and year 2002-‘current’
3. 1 and 2
