# Supplementary material for: Developing an evidence-based clinical pathway for the assessment, diagnosis and management of acute Charcot Neuro-Arthropathy: a systematic review
Source: J Foot Ankle Res. 2013 Jul 30;6:30. doi: 10.1186/1757-1146-6-30 (PMC3737070; doi:10.1186/1757-1146-6-30)
Supplement: Additional file 2 — Level II evidence. [file 1757-1146-6-30-S2.doc]

**Additional file 2 - Level II e**vidence

|  | **Author** | **Title** | **Country of publication** | **Study focus** | **Study design /**  **Population/ Sample size** | **Intervention/**  **control** | **Primary outcome** | **Result** |
| --- | --- | --- | --- | --- | --- | --- | --- | --- |
| 1 | Pakarinen TK, Laine HJ, Maenpaa H, Mattila P, Lahtela J. (2011) | The effect of zoledronic acid on the clinical resolution of Charcot neuroarthropathy: a pilot randomized controlled trial. | USA | Acute CN Mx | RCT/  Patients with acute CN/  N = 35 | 3 intravenous infusions of 4 mg zoledronic acid, N = 18/  placebo, N = 17 | Clinical resolution of acute CN determined by total immobilization time (casting plus orthosis) | In the zoledronic acid group, the median time for total immobilization was 27 weeks (range 10**–**62), and in the placebo group it was 20 weeks (20-52) (p=0.02) |
| 2 | Bem R, Jirkovska A, Fejfarova Vm Skibova J, Jude EB. (2006) | [Intranasal calcitonin in the treatment of acute Charcot neuroosteoarthropathy: a randomized controlled trial.](https://ovidsp-tx-ovid-com.cknservices.dotsec.com/sp-3.5.1a/ovidweb.cgi?&S=NCAIFPLOLCDDIODMNCPKFEJCKNFCAA00&Complete+Reference=S.sh.37|190|1) | USA | Acute CN Mx | RCT/  Patients with acute  CN/  N = 32 | Salmon calcitonin nasal spray 200 IU daily with calcium supplementation, N = 16/ calcium supplementation only, N = 16 | Bone metabolism (COOH-terminal telopeptide region of type 1 collagen (1CTP) and bone-specific alkaline phosphatase (BALP)) and disease activity during a 6-month treatment period | The study group had significantly greater reduction in 1CTP in comparison with the control group during the first 3 months. Significant reduction of BALP was seen in the study group at 3 months in comparison with the control group |
| 3 | Pitocco D, Ruotolo V, Caputo S, Mancini L, Collina CM, Manto A, Caradonna P, Ghirlanda G. (2005) | [Six-month treatment with alendronate in acute Charcot neuroarthropathy: a randomized controlled trial.](https://ovidsp-tx-ovid-com.cknservices.dotsec.com/sp-3.5.1a/ovidweb.cgi?&S=PFPJFPOMAEDDIOLENCPKKHFBFFDLAA00&Complete+Reference=S.sh.37|212|1) | USA | Acute CN Mx | RCT/  Patients with a new diagnosis of acute  painful CN/  N = 20 | 70 mg alendronate by mouth once a week for 6 months, N = 11/  placebo, N = 9 | Bone metabolism and  disease process during  a 6-month treatment  period | ICTP did not show significant difference between the two groups at  the outset, but after 6 months, the test group showed a significant decrease. In the test group, hydroxyprolin followed the same trend. Bone alkaline phosphatase reduction was almost significant. Dual-energy X-ray absorptiometry demonstrated an improvement in total foot mineralization and in the distal phalanxes in the test group that had an improvement of the mineralization of the femur. VAS score for pain was significantly improved in the test group. No significant changes were evident in the control group |
