# Supplementary material for: Developing an evidence-based clinical pathway for the assessment, diagnosis and management of acute Charcot Neuro-Arthropathy: a systematic review
Source: J Foot Ankle Res. 2013 Jul 30;6:30. doi: 10.1186/1757-1146-6-30 (PMC3737070; doi:10.1186/1757-1146-6-30)
Supplement: Additional file 3 — Level III-IV evidence. [file 1757-1146-6-30-S3.doc]

**Additional file 3 - Level III-IV e**vidence

|  | **Author** | **Title** | **Country of publication** | **Study focus** | **Study design /**  **Population/ Sample size** | **Primary outcome** | **Result** |
| --- | --- | --- | --- | --- | --- | --- | --- |
| 1 | Christensen TM, Gade-Rasmussen B, Pedersen LW, Hommel E, Holstein PE, Svendsen OL. (2012) | Duration of off-loading and recurrence rate in Charcot osteo-arthropathy treated with less restrictive regimen with rem`ovable walker | Netherlands | Clinical Signs/Symptoms  Clinical Ax  Acute CN Mx  Chronic CN Dx  Long Term Mx | Retrospective case series (IV)/  Patients with diabetes and acute CN/  N = 56 | The influence of duration of offloading on the risk of recurrence of CN and required re-casting | The duration of off-loading for all patients was 141±21 days (mean ± SD). Three patients (5%) were re-casted immediately for exacerbation after re-load and 7 patients (12 %) after recurrence of the CN. Duration of re-casting was 79±44 days. The primary period of off-loading was not statistically significantly different for those not requiring versus those requiring re-casting: 142±24 days compared to 134±41 days |
| 2 | Game FL, Catlow R, Jones GR, Edmonds ME, Jude EB, Rayman G, Jeffcoate WJ. (2012) | Audit of acute Charcot's disease in the UK: the CDUK study | Germany | Clinical Ax  Acute CN Mx  Long Term Mx | Case series (IV)/  Patients with acute CN/  N = 288 | Factors associated with the development and resolution of acute CN | 36% of patients recalled an episode of relevant trauma in the preceding 6 months, while 12% had had surgery to the affected foot. In 101 (35%) cases, ulceration was present at registration and 20% of these had osteomyelitis. Non-removable off-loading devices were used at presentation in 35.4% of cases, with removable off-loading used in 50%. The median time to resolution was 9 months in patients whose initial management included the use of non-removable off-loading, compared with 12 months in the remainder (p=0.001). The median time to resolution in patients who received bisphosphonates (25.4%) was 12 months and was longer than in those who did not (10 months, p=0.005) |
| 3 | Moura-Neto A, Fernandes TD, Zantut-Wittmann DE, Trevisan RO, Sakaki MH, Santos AL, Nery M, Parisi MC. (2012) | [Charcot foot: skin temperature as a good clinical parameter for predicting disease outcome](http://www.ncbi.nlm.nih.gov/pubmed/22296852) | Netherlands | Clinical Ax  Chronic CN Dx Criteria | Case series (IV)/  Patients with diabetes presenting with acute CN/  N = 28 | The effectiveness of inter-limb temperature difference as a clinical parameter of outcome in a group of patients with acute CN in the foot | Skin temperature difference is a good clinical marker for immobilisation withdrawal in acute diabetic Charcot’s disease as the relapse rate was zero when T difference decreases below 2°C. |
| 4 | Richard JL, Almasri M, Schuldiner S. (2012) | [Treatment of acute Charcot foot with bisphosphonates: a systematic review of the literature](http://www.ncbi.nlm.nih.gov/pubmed/22361982) | Germany | Acute CN Mx | Systematic Review of Clinical Trials and Case Series (IV)/  All articles published between 1990-2011 and relating to the treatment of CN with  Bisphosphonates (BPP)/  N = 10 articles | Clinical assessment, changes in bone turnover markers, bone mineral density and radiological assessment | On theoretical grounds, BPPs may have clinical benefit, but the results of published studies are inconclusive. On balance, treatment with BPPs appears rather ineffective and even deleterious for the resolution time of the acute stage; moreover, data on long-term outcomes are not available. There is, therefore, currently little evidence to support the use of BPPs as part of the routine management of patients with diabetes complicated by acute CNO. This is in agreement with the ADA consensus report that suggests that off-loading and immobilsation remain the mainstay of treatment |
| 5 | Sämann A, Pofahl S, Lehmann T, Voigt B, Victor S, Möller F, Müller UA, Wolf G. (2012) | [Diabetic Nephropathy but not HbA1c is Predictive for Frequent Complications of Charcot Feet - Long-term follow-up of 164 Consecutive Patients with 195 Acute Charcot Feet](http://www.ncbi.nlm.nih.gov/pubmed/22421981) | Germany | Clinical Ax  Acute CN Mx  Serology Referral  Long Term Mx | Retrospective case series (IV)/  Patients with acute CN/  N = 164 | Clinical characteristics, treatment and long-term outcome of patients with acute CN | Type 1 vs. Type 2: Age: 43.7 ± 10.9 vs. 57.9 ± 8.9 years (p < 0.001), male gender: 66.7 vs. 77.3 %, diabetes duration: 19.2 ± 9.1 vs. 13 ± 8.6 years (p=0.018) immobilsation for 6 ± 4.2 vs. 5.4 ± 4.5 months, orthopaedic/adjusted shoes: 27.3 vs. 20.5 %, foot surgery: 11.8 vs. 18.2 %. Major complications: 50 vs. 56% (rocker bottom deformities: 23.5 vs. 46.3 %, foot ulcerations: 17.6 vs. 24.6 %, CF amputations: 0 vs. 6 %), not CF amputations: 16.7 vs. 15.3 %, second episodes of CF: 41.6 vs. 18.3 % after 5–132 months. Diabetic nephropathy was associated with an increase of complications |
| 6 | Wu T, Chen PY, Chen CH, Wang CL. (2012) | Doppler spectrum analysis: a potentially useful diagnostic tool for planning the treatment of patients with Charcot arthropathy of the foot? | UK | Clinical Ax | Case Series (IV)/  Patients with acute diabetic CN of the foot and ankle/  N = 15 | Doppler spectrum analysis of the first dorsal metatarsal artery over the course of CN | The Doppler spectra in the unaffected limbs were triphasic in pattern, whereas those in limbs with active Charcot arthropathy showed monophasic forward flow. They returned to normal after a mean of 13.6 weeks (6 to 20) of immobilisation |
| 7 | Christensen TM, Simonsen L, Holstein PE, Svendsen OL, Bulow J. (2011) | Sympathetic neuropathy in diabetes mellitus patients does not elicit Charcot osteoarthropathy | Netherlands | Clinical Signs/Symptoms  Clinical Ax  Criteria for Dx | Case Control Study (III-2)/  Patients with diabetes mellitus with or without CN/  N = 49 | Degree of neuropathy (autonomic and somatic) in patients with diabetes mellitus with or without CN | The patients with acute Charcot foot and first toe amputation had an increased blood flow in the affected foot and weakened but not absent venoarteriolar sympathetic axon reflex. In the other patient groups, a normal venoarteriolar sympathetic axon reflex in the feet was found |
| 8 | Pickwell KM. van Kroonenburgh MJ. Weijers RE. van Hirtum PV. Huijberts MS. Schaper NC. (2011) | F-18 FDG PET/CT Scanning in Charcot Disease: A Brief Report | USA | Clinical Ax  Imaging referral  Acute Dx Criteria | Case Series (IV)/  Patients with CN/  N =10 | Investigating the relation between bony abnormalities and the (concurrent) inflammatory response in acute CN using F-18 FDG PET/CT scanning | Nine patients had increased uptake of F-18 FDG, indicating inflammation, in 25 areas of soft tissue and/or bone without concurrent bony abnormalities on CT |
| 9 | Wukich DK, Sung W, Wipf SAM, Armstrong DG. (2011) | The consequences of complacency: managing the effects of unrecognized Charcot feet | UK | Clinical Signs/Symptoms  Clinical Ax  Imaging referral  Acute Dx Criteria  DDx | Retrospective Cohort Study (III-2)/  Patients with stage 0 CN/  N = 20 | Complications, including ulcer formation, infection, progression into active CN and the need for surgical reconstruction | The diagnosis of Charcot neuroarthropathy was missed in 19 of 20 patients prior to referral. The average delay in treatment in Group I was 4.1+/-0.7 weeks compared with 8.7+/-6.8 weeks in Group II (Mann–Whitney U-test = 24.5, n1 = 15, n2 = 7, P < 0.05 two-tailed). Sixteen of 22 feet (72%) developed a complication during their treatment course. Group II experienced more complications than Group I (66.7% compared with 14.3%). Overall, eleven of 22 feet (50%) required surgical treatment; however, no patient required an amputation during the follow-up course |
| 10 | Zampa V, Bargellini I, Rizzo L, Turini F, Ortori S, Piaggesi A, Bartolozzi C. (2011) | Role of dynamic MRI in the follow-up of acute Charcot foot in patients with diabetes mellitus | Germany | Clinical signs/symptoms  Clinical Ax  Imaging referral  Acute CN dx criteria  Acute Mx  Long Term Mx | Case Series (III-3)/  Diabetic patients with acute CN/  N= 40 | Magnetic resonance imaging (MRI) in assessing the level of activity of acute  Charcot foot, monitoring treatment response and predicting healing time | At baseline, mean contrast medium uptake rate was 136±49.7% and the mean SI ratio was 5±3. At 3 months’ follow-up, reduction of the contrast medium uptake rate was observed in all patients with improved clinical findings (n=34), whereas the SI ratio was reduced in 15/34 (44.1%) patients. Mean healing time was significantly related to the baseline contrast medium uptake rate (P=0.005); it was 5.3±2.7 months in patients with contrast medium uptake rate **≤**100%, compared with 9.1±2.5 months in the remaining patients (P=0.0003) |
| 11 | Bem R, Jirkovska A, Dubsky M, Fejfarova V, Buncova M, Skibova J, Jude EB. (2010) | [Role of quantitative bone scanning in the assessment of bone turnover in patients with Charcot foot.](https://ovidsp-tx-ovid-com.cknservices.dotsec.com/sp-3.5.1a/ovidweb.cgi?&S=KHIGFPGMKJDDIOMENCPKFAOBDLLOAA00&Complete+Reference=S.sh.42|177|1) | USA | Clinical Ax  Imaging referral  Chronic CN Dx Criteria | Case Control (III-2)/  Patients with acute and non-acute CN/  N = 42 | Quantitative bone scan parameters as markers of CN activity | Significant correlations between quantitative bone scan parameters and bone turnover markers were observed (all P _ 0.05). These parameters decreased after treatment of CNO, and its reduction to the baseline value correlated with differences of bone turnover markers and STD (all P< 0.05) |
| 12 | Christensen TM, Bulow J, Simonsen L, Holstein PE, Svendsen OL. (2010) | [Bone mineral density in diabetes mellitus patients with and without a Charcot foot.](https://ovidsp-tx-ovid-com.cknservices.dotsec.com/sp-3.5.1a/ovidweb.cgi?&S=KHIGFPGMKJDDIOMENCPKFAOBDLLOAA00&Complete+Reference=S.sh.42|176|1) | Scandinavia | Clinical Ax  Acute CN dx criteria | Case Control (III-2)/  Patients with diabetes/  N = 49 | Bone mineral density in patients with diabetes mellitus and CN | There was an increase in the markers of bone turnover in the patients with acute CA. The BMD of the calcaneus was statistically lower in the affected foot in patients with chronic Charcot (P <0.01), than in the unaffected foot, but there were no statistically significant differences between the BMD of the calcaneus in the feet in the other groups |
| 13 | Press R. (2010) | [Conservative treatment can be efficacious in treatment of Charcot arthropathy](http://web.ebscohost.com.ezproxy.library.uq.edu.au/ehost/viewarticle?data=dGJyMPPp44rp2%2FdV0%2Bnjisfk5Ie46a9It62zT6%2Bk63nn5Kx95uXxjL6urVGtqK5JsJa1UrCquE2vls5lpOrweezp33vy3%2B2G59q7SbKqr1CzqLVOspzqeezdu33snOJ6u9nnhrCmpIzf3btZzJzfhruorkmurrJLtqu0PuTl8IXf6rt%2B8%2BLqjOPu8gAA&hid=113) | USA | Acute CN mx  LT CN mx  Discussion | Retrospective case series (IV)/  Patients with CN of the foot and ankle/  N = 340 | Nature history and long term outcomes of CN treatments | Mean number of surgeries per patient was 1.21, but 159 (46%) required no surgical intervention. Equal rates in unilateral and bilateral cases.  Partial foot and below-knee amputations consisted of less than 5% of all surgeries. 331 (97.4%) patients displayed ambulatory ability at their last visit |
| 14 | Basu S, Zhuang H, Alavi A. (2012) | [FDG PET and PET/CT imaging in complicated diabetic foot](https://ovidsp-tx-ovid-com.cknservices.dotsec.com/sp-3.5.1a/ovidweb.cgi?&S=KHIGFPGMKJDDIOMENCPKFAOBDLLOAA00&Complete+Reference=S.sh.42|133|1) | USA | Imaging referral | Case series (IV)/  Patients with CN/  N = 39 | Diagnostic outcomes of CN using FDG-PET versus MRI | The sensitivity and accuracy of FDG-PET in the diagnosis of Charcot neuroarthropathy were 100% and 93.8%, respectively, and for MRI the values were 76.9% and 75.0%, respectively. These results indicated the valuable role of FDG-PET in the setting of Charcot neuroarthropathy by reliably differentiating it from osteomyelitis both in general and when a foot ulcer is present |
| 15 | [Leung HB](https://ovidsp-tx-ovid-com.cknservices.dotsec.com/sp-3.5.1a/ovidweb.cgi?&S=NCAIFPLOLCDDIODMNCPKFEJCKNFCAA00&Search+Link="Leung+HB".au.), [Ho YC](https://ovidsp-tx-ovid-com.cknservices.dotsec.com/sp-3.5.1a/ovidweb.cgi?&S=NCAIFPLOLCDDIODMNCPKFEJCKNFCAA00&Search+Link="Ho+YC".au.), [Wong WC](https://ovidsp-tx-ovid-com.cknservices.dotsec.com/sp-3.5.1a/ovidweb.cgi?&S=NCAIFPLOLCDDIODMNCPKFEJCKNFCAA00&Search+Link="Wong+WC".au.). (2009) | Charcot foot in a Hong Kong Chinese diabetic population | Hong Kong | Clinical Ax  Serology referrals  Imaging referrals  Acute Mx  Long Term Mx | Retrospective cohort study (III-3)/  Diabetic patients with CN/  N = 25 | Clinical presentations between patients with CN and the controls | Mean age was 59 (standard deviation, 14; range, 38-85) years, diabetes diagnosed for a mean of 11 (standard deviation, 8; range, 0-30) years. No patient had peripheral vascular disease. Delayed presentation occurred in 11 patients. Presentation was usually unilateral. In the minority (n=3, 12%) with bilateral involvement, presentation was sequential. CN affected the mid-foot in 64% of the patients. Superimposed infection was common (61%).  Only one patient underwent major amputation |
| 16 | Pakarinen TK, Laine HJ, Maenpaa H, Mattila P, Lahtela J. (2009) | [Long-term outcome and quality of life in patients with Charcot foot.](https://ovidsp-tx-ovid-com.cknservices.dotsec.com/sp-3.5.1a/ovidweb.cgi?&S=NCAIFPLOLCDDIODMNCPKFEJCKNFCAA00&Complete+Reference=S.sh.37|84|1) | Netherlands | Introduction  Clinical Ax  Acute CN Dx Criteria  Serology referrals  Chronic CN dx  Long Term Mx | Cross sectional (IV)/  Patients with CN/  N = 41 | Nature history and long term outcomes of CN treatments | Overall mortality rate was 29% (12/41), 67% of CN feet suffered at least one episode of ulceration and 50% (15/30) of affected feet had surgical treatment resulting in 29 operations. The need for surgical management increased markedly 4 years after the diagnosis. Correct diagnosis within 3 months resulted in better functional outcome |
| 17 | Bevan WP. Tomlinson MP. (2008) | [Radiographic measures as a predictor of ulcer formation in diabetic Charcot midfoot](https://ovidsp-tx-ovid-com.cknservices.dotsec.com/sp-3.5.1a/ovidweb.cgi?&S=NCAIFPLOLCDDIODMNCPKFEJCKNFCAA00&Complete+Reference=S.sh.37|133|1) | USA | Chronic CN dx criteria  Long Term Mx | Retrospective case series (IV)/  Patients with diabetic CN/  N = 19 | Does the magnitude of midfoot deformity, measured using radiographic criteria, predicts risk of ulceration in CN | When radiographic measures of feet with and without midfoot skin pathology were compared, the lateral  talar-first metatarsal angle was significantly associated with skin pathology (p < 0.001) |
| 18 | de Souza LJ. (2008) | [Charcot arthropathy and immobilization in a weight-bearing total contact cast](https://ovidsp-tx-ovid-com.cknservices.dotsec.com/sp-3.5.1a/ovidweb.cgi?&S=NCAIFPLOLCDDIODMNCPKFEJCKNFCAA00&Complete+Reference=S.sh.37|139|1) | USA | Acute Mx  Long Term Mx | Case series (IV)/  Patients with CN of the foot or ankle/  N = 27 | Treatment outcomes after managing acute CN with a weight-bearing total contact casts | No deleterious effects from weight-bearing, specifically with regard to skin ulceration or rapid deterioration of osseous architecture, was observed in 33 of the 34 feet |
| 19 | Judge MS. (2008) | [Infection and neuroarthropathy: the utility of C-reactive protein as a screening tool in the Charcot foot](https://ovidsp-tx-ovid-com.cknservices.dotsec.com/sp-3.5.1a/ovidweb.cgi?&S=NCAIFPLOLCDDIODMNCPKFEJCKNFCAA00&Complete+Reference=S.sh.37|147|1) | USA | Serology referral  Ddx | Case series (IV)/  Patients with diabetes and acute CN/  N = 7 | C-reactive protein to aid in the diagnosis of infection in acute CN | C-reactive protein level was not suggestive of infection, and none of the patients progressed to a diagnosis of infection |
| 20 | Schlossbauer T. Mioc T. Sommerey S. Kessler SB. Reiser MF. Pfeifer KJ. (2008) | [Magnetic resonance imaging in early stage charcot arthropathy: correlation of imaging findings and clinical symptoms](https://ovidsp-tx-ovid-com.cknservices.dotsec.com/sp-3.5.1a/ovidweb.cgi?&S=NCAIFPLOLCDDIODMNCPKFEJCKNFCAA00&Complete+Reference=S.sh.37|126|1) | Germany | Imaging referral | Case series (IV)/  Patients with CN/  N = 13 | Qualitative and quantitative MRI findings in early stage CN and correlation with clinical symptoms | Bone marrow oedema in affected bones significantly decreased (p<0.001). Soft tissue oedema and pain showed a significant correlation with intensity of bone marrow oedema (p<0.05). The presence of bone marrow oedema in the STIR sequence was strongly associated with a corresponding contrast enhancement (p<0.001) |
| 21 | Sinacore DR, Hastings MK, Bohnert KL, Fielder FA, Villareal DT, Blair VP 3rd, Johnson JE. (2008) | [Inflammatory osteolysis in diabetic neuropathic (charcot) arthropathies of the foot](https://ovidsp-tx-ovid-com.cknservices.dotsec.com/sp-3.5.1a/ovidweb.cgi?&S=NCAIFPLOLCDDIODMNCPKFEJCKNFCAA00&Complete+Reference=S.sh.37|123|1) | USA | Clinical Ax | Case Control (III-2)/  Patients with diabetes, peripheral neuropathy and CN of the foot or ankle/  N = 96 | Tarsal BMD associated with acute inflammation (i.e. inflammatory osteolysis) in individuals with chronic diabetes mellitus (DM), peripheral neuropathy (PN), and recent-onset neuropathic (Charcot) arthropathy (NCA) of the foot | Skin temperature differences averaged 6.7°F (SD**+/-**4.0°F) (involved foot minus noninvolved foot) in the feet of the subjects with DM, PN, and NCA compared with 0.0°F (SD**+/-**1.3°F) in the feet of the control subjects. Calcaneal BMD averaged 384 mg/cm2 (SD**+/-**110) in the involved feet and 467 mg/cm2 (SD**+/-**123) in the noninvolved feet of the subjects with DM, PN, and NCA and 545 mg/cm2 (SD**+/-**121) in combined right and left feet of the control subjects |
| 22 | Stuck RM, Sohn MW, Budiman-Mak E, Lee TA, Weiss KB. (2008) | [Charcot arthropathy risk elevation in the obese diabetic population](https://ovidsp-tx-ovid-com.cknservices.dotsec.com/sp-3.5.1a/ovidweb.cgi?&S=NCAIFPLOLCDDIODMNCPKFEJCKNFCAA00&Complete+Reference=S.sh.37|125|1) | USA | Clinical Ax  Serology referral | Case series (IV)/  DVA patients with diabetes/  N = 652 | Association of obesity, peripheral neuropathy, and other risk factors with the CN incidence rate in a large diabetic population | 652 (0.12%) were newly diagnosed with CN in 2003. Compared with persons without obesity or peripheral neuropathy, those with obesity alone were approximately 59% more likely, those with neuropathy alone were 14 times more likely, and those with both obesity and neuropathy were 21 times more likely to develop Charcot arthropathy. Ages  55 to 64 years, diabetes duration 6 years or more, hemoglobin-A1c 7% or more, renal failure, arthritis, and deficiency anemia also were associated with an increased incidence of Charcot arthropathy |
| 23 | Verity S, Sochocki M, Embil JM, Trepman E. (2008) | [Treatment of Charcot foot and ankle with a prefabricated removable walker brace and custom insole](https://ovidsp-tx-ovid-com.cknservices.dotsec.com/sp-3.5.1a/ovidweb.cgi?&S=NCAIFPLOLCDDIODMNCPKFEJCKNFCAA00&Complete+Reference=S.sh.37|117|1) | Netherlands | Acute CN mx  Long Term Mx | Case series (IV)/  Patients with acute CN/  N = 21 | Management outcomes and disease process using prefabricated removable walker brace and custom insole | 17 (68%) feet and ankles had consolidation (stage III) of the Charcot arthropathy (average duration of brace use, 29+/-19 weeks) and were subsequently treated with rocker sole shoes, insoles, and ankle foot orthoses; 8 (32%) feet and ankles had ongoing brace treatment. Three feet developed new deformity during brace treatment, but average radiographic parameters of hindfoot to forefoot alignment had minimal change between initial and final radiographs at an average of 36-24 weeks after initial radiographic evaluation |
| 24 | Basu S, Chryssikos T, Houseni M, Malay DS, Shah J, Zhuang H, Alavi A. (2007) | [Potential role of FDG PET in the setting of diabetic neuro- osteoarthropathy: Can it differentiate uncomplicated Charcot's neuroarthropathy from osteomyelitis and soft-tissue infection?](https://ovidsp-tx-ovid-com.cknservices.dotsec.com/sp-3.5.1a/ovidweb.cgi?&S=NBDAFPIKLFDDIOFONCPKKHIBKLCDAA00&Link+Set=S.sh.38|206|sl_10) | USA | Imaging referral | Case Control Study (III-2)/  Patients with diabetes (N=43) or patients without diabetes (N=20)/  N = 63 | To investigate the potential utility of FDG PET imaging in the setting of acute CN | The results support a valuable role of FDG PET in the setting of Charcot’s neuroarthropathy by reliably differentiating it from osteomyelitis both in general and when foot ulcer is present |
| 25 | Kimmerle R, Chantelau E. (2007) | [Weight-bearing intensity produces Charcot deformity in injured neuropathic feet in diabetes](https://ovidsp-tx-ovid-com.cknservices.dotsec.com/sp-3.5.1a/ovidweb.cgi?&S=NCAIFPLOLCDDIODMNCPKFEJCKNFCAA00&Complete+Reference=S.sh.37|166|1) | Germany | Acute CN mx | Retrospective Cohort Study (III-2)/  Patients with diabetic neuropathy and a history of a non fracture injury/  N = 34 | Investigate the relationship between the intensity of unrestrained weight bearing after a non fracture injury (i.e. sprain) and the development of acute CN in patients with diabetic neuropathy | Unrestrained weight bearing of injured foot bones and joints of more than 400kg x week prompts Charcot deformities, with disintegration of the Lanfranc’s joint. Early offloading by TCC treatment allows healing without deformities |
| 26 | Masding M, Spruce M, Coppini D. (2007) | [A retrospective case study of Charcot osteoarthropathy](http://web.ebscohost.com.ezproxy.library.uq.edu.au/ehost/viewarticle?data=dGJyMPPp44rp2%2FdV0%2Bnjisfk5Ie46a9It62zT6%2Bk63nn5Kx95uXxjL6urVGtqK5JsJa0UrKvuEmwls5lpOrweezp33vy3%2B2G59q7SbKqr1CzqLVOspzqeezdu33snOJ6u9nnhrCmpIzf3btZzJzfhruorki3rbVOr6quPuTl8IXf6rt%2B8%2BLqjOPu8gAA&hid=113) | UK | Clinical Ax  Acute CN dx criteria | Retrospective Case Series (IV)/  Patients with CN/  N = 15 | Characteristics and risk markers of patients with CN | Patients with CN had significantly longer mean duration of diabetes, higher mean HbA1c levels, less prevalence of PVD |
| 27 | [Petrova NL](http://www.ncbi.nlm.nih.gov/pubmed?term=Petrova NL%5BAuthor%5D&cauthor=true&cauthor_uid=17392560), [Moniz C](http://www.ncbi.nlm.nih.gov/pubmed?term=Moniz C%5BAuthor%5D&cauthor=true&cauthor_uid=17392560), [Elias DA](http://www.ncbi.nlm.nih.gov/pubmed?term=Elias DA%5BAuthor%5D&cauthor=true&cauthor_uid=17392560), [Buxton-Thomas M](http://www.ncbi.nlm.nih.gov/pubmed?term=Buxton-Thomas M%5BAuthor%5D&cauthor=true&cauthor_uid=17392560), [Bates M](http://www.ncbi.nlm.nih.gov/pubmed?term=Bates M%5BAuthor%5D&cauthor=true&cauthor_uid=17392560), [Edmonds ME](http://www.ncbi.nlm.nih.gov/pubmed?term=Edmonds ME%5BAuthor%5D&cauthor=true&cauthor_uid=17392560). (2007) | Is there a Systemic Inflammatory Response in the Acute Charcot Foot? | USA | Clinical Ax  Serology referral  Imaging referral | Case Series (IV)/  Patients with acute CN/  N = 36 | Systemic serologic markers of inflammation in acute CN | There were 21 type 1 (11 male and 10 female) and 15 type 2 (7male and 8 female) diabetic patients. Median age was 51 years (41– 62), and median duration of diabetes was 20 years (13– 26.5). Skin foot temperature was 3.1°C  (2.4 – 4.2) greater in the Charcot foot compared with the contralateral foot. Median CRP level was 5.8 mg/l (5–11) and <5 mg/l in 47.2% of patients presenting with acute Charcot osteoarthropathy. Median  ESR was 21 mm/h (13–36); WCC was 7.0 (5.8 – 8.1), reference range 4–11; GHb was 8.5% (7.3–10.3), reference range **<**6; and Hb was 13.1 g/dl (11.7–14.7). The median creatinine level was 88mol/l (79 –109), reference range 45–120, and only one patient had renal failure and was on hemodialysis treatment |
| 28 | Pinzur MS, Lio T, Posner M. (2006) | [Treatment of Eichenholtz stage I Charcot foot arthropathy with a weightbearing total contact cast](https://ovidsp-tx-ovid-com.cknservices.dotsec.com/sp-3.5.1a/ovidweb.cgi?&S=NCAIFPLOLCDDIODMNCPKFEJCKNFCAA00&Complete+Reference=S.sh.37|191|1) | USA | Clinical Ax  Acute CN Mx  Long Term Mx | Case Series (IV)/  Patients with acute CN (Eichenholtz  stage I)/  N = 9 | The success rate of total contact casting in a small group of patients and to establish a benchmark time period for treatment | All subjects were able to use commercially available depth-inlay shoes and custom accommodative foot orthoses at an average of 9.2 (range 8 to 16) weeks. One subject developed a superficial ulcer that resolved with footwear modification |
| 29 | Chantelau E. (2005) | [The perils of procrastination: effects of early vs. delayed detection and treatment of incipient Charcot fracture](https://ovidsp-tx-ovid-com.cknservices.dotsec.com/sp-3.5.1a/ovidweb.cgi?&S=NCAIFPLOLCDDIODMNCPKFEJCKNFCAA00&Link+Set=S.sh.37|197|sl_10) | UK | Clinical Signs/Symptoms  Imaging referrals  Acute CN Mx  Acute CN dx criteria  Negative Dx  Chronic CN dx criteria  DDx | Retrospective Case Control Study (III-2)/  Patients with acute CN/  N = 24 | Extent of foot fractures and of foot deformity, respectively, observed after healing  (Eichenholtz stage III) of the CN foot | In 19 of the 24 patients, CN had been misdiagnosed prior to referral. Additional imaging techniques (MRI, CT scan or bone scintigraphy) had been performed in 10 patients prior to referral. While these techniques had been used more frequently in the cases vs. the controls (  P = 0.012), misdiagnosis was less frequent in the cases vs. the controls (P =0.013). Only one out of 11 case patients developed extended foot fractures and severe deformity, vs. 12 out of 13 control patients (P <0.001) |
| 30 | Petrova NL, Foster AV, Edmonds ME. (2005) | [Calcaneal bone mineral density in patients with Charcot neuropathic osteoarthropathy: differences between Type 1 and Type 2 diabetes](https://ovidsp-tx-ovid-com.cknservices.dotsec.com/sp-3.5.1a/ovidweb.cgi?&S=PFPJFPOMAEDDIOLENCPKKHFBFFDLAA00&Link+Set=S.sh.37|209|sl_10) | UK | Clinical Ax  Acute CN dx criteria | Case Control Study (III-2)/  Patients with Type 1 or Type 2 diabetes/  N = 130 | Bone mineral density in Type 1 and Type 2 patients with CN compared with Type 1 and Type 2 diabetic control patients of a similar age and duration of diabetes | Bone density was reduced in the non-CN in Type 1 but not in Type 2 diabetes. Type 2 patients had high temperature and vibration thresholds in contrast to Type 1 patients who had a high temperature threshold only |
| 31 | Saltzman CL, Hagy ML, Zimmerman B, Estin M, Cooper R. (2005) | [How effective is intensive non-operative initial treatment of patients with diabetes and Charcot arthropathy of the feet?](https://ovidsp-tx-ovid-com.cknservices.dotsec.com/sp-3.5.1a/ovidweb.cgi?&S=KHIGFPGMKJDDIOMENCPKFAOBDLLOAA00&Link+Set=S.sh.42|71|sl_10) | USA | Introduction  Clinical Ax  Acute CN Mx  Long Term Mx | Retrospective Case Series (IV)/  Patients with diabetes mellitus associated CN/  N = 115 | Rates of below-knee amputation, long-term brace wear, reulceration, reconstructive surgeries, and bilaterally for patients treated in a structured, intensive, and non-operative manner | 2.7% annual rate of amputation, a 23% risk of requiring bracing for more than 18 months, and a 49% risk of recurrent ulceration |
| 32 | Anderson JJ, Woelffer KE. Holtzman JJ. Jacobs AM. (2004) | [Bisphosphonates for the treatment of Charcot neuroarthropathy](https://ovidsp-tx-ovid-com.cknservices.dotsec.com/sp-3.5.1a/ovidweb.cgi?&S=PFPJFPOMAEDDIOLENCPKKHFBFFDLAA00&Complete+Reference=S.sh.37|222|1) | USA | Acute CN Dx criteria  Acute CN Mx | Case Control Study (III-2)/  Patients diagnosed with acute CN/  N = 23 | Disease process and resolution of acute CN | After pamidronate infusion, limb temperature decreased a mean 2.8°F by 48 hours and 7.4°F by 2 weeks. The alkaline phosphatase levels also decreased an average 53% 2 weeks after infusion. The control group showed no reduction in limb temperature at 48 hours, and had an average limb temperature reduction of 2.3°F at 2 to 3 weeks. This was significantly less than the temperature reduction in the treated group (P = .008 at 48 hours and P = .001 at 2 weeks). Mean alkaline phosphatase levels declined only 9% in the control group, a significantly smaller decline than in the pamidronate-infusion group (P = .001). These results suggest that pamidronate may be useful in halting the acute phase of CN |
| 33 | Foltz KD, Fallat LM, Schwartz S. (2004) | [Usefulness of a brief assessment battery for early detection of Charcot foot deformity in patients with diabetes](https://ovidsp-tx-ovid-com.cknservices.dotsec.com/sp-3.5.1a/ovidweb.cgi?&S=PFPJFPOMAEDDIOLENCPKKHFBFFDLAA00&Complete+Reference=S.sh.37|233|1) | USA | Clinical Ax  Acute CN Mx  Ddx | Case Control Study (III-2)/  Patients with diabetes and chronic CN or CN-free/  N = 59 | Historical and physical findings of patients with established CN compared to a heterogeneous CN-free population of patients with diabetes | The results indicate that simple neurologic testing combined with a thorough patient history were the most beneficial tools to determine diabetics with a higher probability of developing CN |
| 34 | Bitsch M, Saunte D.M. Dall C, Holstein P.E. (2003) | [Charcot's arthropathy following digital amputation in the diabetic foot](https://ovidsp-tx-ovid-com.cknservices.dotsec.com/sp-3.5.1a/ovidweb.cgi?&S=KHIGFPGMKJDDIOMENCPKFAOBDLLOAA00&Complete+Reference=S.sh.42|85|1) | Netherlands | Clinical Ax  Imaging referral  Negative Dx | Retrospective Case Series (IV)/  Patients with diabetes/  N = 126 | The onset of CN following digital amputation in patients with diabetes | The incidence of CN in patients with normal peripheral perfusion was found to be 23% (7/30 patients) following trans-metatarsal amputation of the first toe and occurred only in patients with normal peripheral perfusion and neuropathy. No cases of Charcot were recorded in patients with vascular insufficiency, which had a partial amputation of the first toe or if only one or more of the small toes were amputated. The onset of CN was within 3 month after surgery in five cases and after 4 and 6 months in two cases |
| 35 | Pakarinen TK, Laine HJ, Honkonen SE, Peltonen J, Oksala H, Lahtela J. (2002) | [Charcot arthropathy of the diabetic foot. Current concepts and review of 36 cases](https://ovidsp-tx-ovid-com.cknservices.dotsec.com/sp-3.5.1a/ovidweb.cgi?&S=PFPJFPOMAEDDIOLENCPKKHFBFFDLAA00&Complete+Reference=S.sh.37|257|1) | Scandinavia | Introduction  Clinical Ax  Acute CN Mx  Imaging referral  Serology referral  Ddx  Chronic CN dx criteria  Surgical referral | Retrospective Case Series (IV)/  Diabetic patients presenting with CN/  N = 36 | Current diagnostics and treatment of CN | 29 cases were diagnosed in the dissolution stage, 2 in coalescence, and 5 in the resolution stage. The diagnostic delay averaged 29 weeks. Treatment with cast immobilization ranged from 4 to 37 weeks (mean 11 weeks). A total of 14 surgical procedures were carried out on 10 patients |
