# Supplementary material for: Developing an evidence-based clinical pathway for the assessment, diagnosis and management of acute Charcot Neuro-Arthropathy: a systematic review
Source: J Foot Ankle Res. 2013 Jul 30;6:30. doi: 10.1186/1757-1146-6-30 (PMC3737070; doi:10.1186/1757-1146-6-30)
Supplement: Additional file 4 — Level EO evidence. [file 1757-1146-6-30-S4.doc]

**Additional file 4 - Level EO e**vidence

|  | **Author** | **Title** | **Country of publication** | **Study focus** |
| --- | --- | --- | --- | --- |
| 1 | [Baglioni P](https://ovidsp-tx-ovid-com.cknservices.dotsec.com/sp-3.5.1a/ovidweb.cgi?&S=JOOKFPIAPEDDOOFINCPKPHMCDEDOAA00&Search+Link="Baglioni+P".au.), [Malik M](https://ovidsp-tx-ovid-com.cknservices.dotsec.com/sp-3.5.1a/ovidweb.cgi?&S=JOOKFPIAPEDDOOFINCPKPHMCDEDOAA00&Search+Link="Malik+M".au.), [Okosieme OE](https://ovidsp-tx-ovid-com.cknservices.dotsec.com/sp-3.5.1a/ovidweb.cgi?&S=JOOKFPIAPEDDOOFINCPKPHMCDEDOAA00&Search+Link="Okosieme+OE".au.). (2012) | Acute Charcot Foot | UK | Introduction  Clinical Signs/Symptoms  Clinical Ax  Serology referral  Imaging referral  Ddx  Acute CN Mx  Chronic Dx Criteria |
| 2 | Malhotra S, Bello E, Kominsky S. (2012) | [Diabetic foot ulcerations: biomechanics, charcot foot, and total contact cast](http://www.ncbi.nlm.nih.gov/pubmed/22817854) | UK | Surgical referral |
| 3 | Rudrappa S, Game F, Jeffcoate W. (2012) | [Recurrence of the acute Charcot foot in diabetes](http://www.ncbi.nlm.nih.gov/pubmed/22587408) | UK | Clinical Ax  LT Mx |
| 4 | Valabhji J. (2012) | [Foot problems in patients with diabetes and chronic kidney disease](http://www.ncbi.nlm.nih.gov/pubmed/22348369) | Switzerland | Acute CN Mx  Serology referral  Imaging referral  LT Mx |
| 5 | Wünschel M, Wülker N, Gesicki M. (2012) | [Charcot arthropathy of the first metatarsophalangeal joint](http://www.ncbi.nlm.nih.gov/pubmed/22461274) | USA | Clinical Ax  Serology referral |
| 6 | Bramham R, Wraight P, May K. (2011) | Management of charcot neuroarthropathy | UK | Clinical Ax  Acute CN Mx  Eichenholtz Table  Imaging referral  LT Mx  Surgical referral |
| 7 | Chisholm KA, Gilchrist JM. (2011) | [The Charcot Joint: A modern neurologic perspective](https://ovidsp-tx-ovid-com.cknservices.dotsec.com/sp-3.5.1a/ovidweb.cgi?&S=KHIGFPGMKJDDIOMENCPKFAOBDLLOAA00&Complete+Reference=S.sh.42|10|1) | USA | Clinical Signs/Symptoms  Clinical Ax  Imaging referral  Chronic CN Dx Criteria  LT Mx |
| 8 | Gouveri E, Papanas N. (2011) | [Charcot osteoarthropathy in diabetes: A brief review with an emphasis on clinical practice](http://www.ncbi.nlm.nih.gov/pubmed/21691556) | China | Clinical Ax  Acute CN Dx Criteria  Serology referral  Acute CN Mx |
| 9 | Ramanujam CL, Facaros Z. (2011) | [An overview of conservative treatment options for diabetic Charcot foot neuroarthropathy](https://ovidsp-tx-ovid-com.cknservices.dotsec.com/sp-3.5.1a/ovidweb.cgi?&S=KHIGFPGMKJDDIOMENCPKFAOBDLLOAA00&Complete+Reference=S.sh.42|145|1) | USA | Acute CN Mx  LT Mx |
| 10 | Rogers LC, Frykberg RG, Armstrong DG, Boulton AJM, Edmonds M, Van GH, Hartemann A, Game F, Jeffcoate W, Jirkovska A, Jude E, Morbach S, Morrison WB, Pinzur M, Pitocco D, Sanders L, Wukich DK, Uccioli L. (2011) | The Charcot Foot in Diabetes | USA | Clinical Signs/Symptoms  Clinical Ax  Imaging referrals  Acute CN Dx critera  Acute CN Mx  LT Mx  Surgical referral |
| 11 | [Aragon-Sanchez J](https://ovidsp-tx-ovid-com.cknservices.dotsec.com/sp-3.5.1a/ovidweb.cgi?&S=KBKCFPKOFFDDNOMONCPKGCFBBBOCAA00&Search+Link="Aragon-Sanchez+J".au.), [Lazaro-Martinez JL](https://ovidsp-tx-ovid-com.cknservices.dotsec.com/sp-3.5.1a/ovidweb.cgi?&S=KBKCFPKOFFDDNOMONCPKGCFBBBOCAA00&Search+Link="Lazaro-Martinez+JL".au.), [Hernandez-Herrero MJ](https://ovidsp-tx-ovid-com.cknservices.dotsec.com/sp-3.5.1a/ovidweb.cgi?&S=KBKCFPKOFFDDNOMONCPKGCFBBBOCAA00&Search+Link="Hernandez-Herrero+MJ".au.). (2010) | Triggering mechanisms of neuroarthropathy following conservative surgery for osteomyelitis | UK | Clinical Signs/Symptoms  Clinical Ax |
| 12 | [Botek G](https://ovidsp-tx-ovid-com.cknservices.dotsec.com/sp-3.5.1a/ovidweb.cgi?&S=KBKCFPKOFFDDNOMONCPKGCFBBBOCAA00&Search+Link="Botek+G".au.), [Anderson MA](https://ovidsp-tx-ovid-com.cknservices.dotsec.com/sp-3.5.1a/ovidweb.cgi?&S=KBKCFPKOFFDDNOMONCPKGCFBBBOCAA00&Search+Link="Anderson+MA".au.), [Taylor R](https://ovidsp-tx-ovid-com.cknservices.dotsec.com/sp-3.5.1a/ovidweb.cgi?&S=KBKCFPKOFFDDNOMONCPKGCFBBBOCAA00&Search+Link="Taylor+R".au.). (2010) | Charcot neuroarthropathy: An often overlooked complication of diabetes | USA | Clinical Ax  Ddx  Serology referral |
| 13 | Molines L, Darmon P, Raccah D. (2010) | Charcot's foot: newest findings on its pathophysiology, diagnosis and treatment | France | Introduction  Clinical Ax  Imaging referral  Acute CN Mx |
| 14 | Obolensky L, Trimble K. (2010) | [Importance of close surveillance for Charcot arthropathy in diabetic patients presenting to the emergency department with low-energy foot injuries](https://ovidsp-tx-ovid-com.cknservices.dotsec.com/sp-3.5.1a/ovidweb.cgi?&S=KHIGFPGMKJDDIOMENCPKFAOBDLLOAA00&Complete+Reference=S.sh.42|167|1) | UK | Clinical Ax |
| 15 | Pappalardo J, Fitzgerald R. (2010) | Utilization of advanced modalities in the management of diabetic Charcot neuroarthropathy | USA | Clinical Ax  Ddx  Imaging referral  Acute CN Mx |
| 16 | Perrin BM, Gardner MJ, Suhaimi A, Murphy D. (2010) | Charcot osteoarthropathy of the foot | Australia | Introduction  Ddx  Clinical Signs/Symptoms  Imaging referral  Serology referral  Acute CN Mx  LT Mx |
| 17 | Schoots IG, Slim FJ, Busch-Westbroek TE, Maas M. (2010) | Neuro-osteoarthropathy of the foot-radiologist: friend or foe? | USA | Clinical Signs/Symptoms  Clinical Ax  Imaging referral  Serology referral  Ddx |
| 18 | Yablon CM, Duggal N, Wu, JS, Shetty SK, Dawson F, Hochman, MG. (2010) | A review of Charcot neuroarthropathy of the midfoot and hindfoot: what every radiologist needs to know | USA | Clinical Signs/Symptoms  Imaging referral  Serology referral  Ddx |
| 19 | Campanaro NR, Gurr JM, Murray RJ. (2009) | [Percutaneous bone biopsy to distinguish osteomyelitis from charcot osteoarthropathy: two case reports](https://ovidsp-tx-ovid-com.cknservices.dotsec.com/sp-3.5.1a/ovidweb.cgi?&S=NCAIFPLOLCDDIODMNCPKFEJCKNFCAA00&Complete+Reference=S.sh.37|79|1) | USA | Imaging referral |
| 20 | Embil JM, Trepman E. (2009) | [A case of diabetic Charcot arthropathy of the foot and ankle](https://ovidsp-tx-ovid-com.cknservices.dotsec.com/sp-3.5.1a/ovidweb.cgi?&S=NCAIFPLOLCDDIODMNCPKFEJCKNFCAA00&Complete+Reference=S.sh.37|90|1) | UK | Clinical Signs/Symptoms  Ddx  Imaging referral  Acute CN Mx |
| 21 | Jones GR, Lomax GA, Eccles KM, Parikh M, McLaughlin C. (2009) | [Pain: The first stage of Charcot arthropathy](https://ovidsp-tx-ovid-com.cknservices.dotsec.com/sp-3.5.1a/ovidweb.cgi?&S=KHIGFPGMKJDDIOMENCPKFAOBDLLOAA00&Link+Set=S.sh.42|115|sl_10) | UK | Clinical Signs/Symptoms |
| 22 | Landsman A, Zgonis T, Neufeld S, Sella EJ. (2009) | [Hindfoot Charcot](https://ovidsp-tx-ovid-com.cknservices.dotsec.com/sp-3.5.1a/ovidweb.cgi?&S=NCAIFPLOLCDDIODMNCPKFEJCKNFCAA00&Complete+Reference=S.sh.37|85|1) | USA | Acute CN Mx  Imaging referral  LT Mx |
| 23 | Thompson P, Hanson D, Langemo DK, Hunter S, Anderson J. (2009) | [Diabetic foot: Charcot neuropathic osteoarthropathy](https://ovidsp-tx-ovid-com.cknservices.dotsec.com/sp-3.5.1a/ovidweb.cgi?&S=NCAIFPLOLCDDIODMNCPKFEJCKNFCAA00&Complete+Reference=S.sh.37|105|1) | USA | Clinical Signs/Symptoms  Serology referral |
| 24 | van der Ven A, Chapman CB, Bowker JH. (2009) | Charcot neuroarthropathy of the foot and ankle | USA | Clinical Ax  Imaging referral  Acute CN Mx |
| 25 | Wipf SAM, Sung W, Wukich DK. (2009) | [An ounce of prevention: recognizing stage 0 charcot](http://web.ebscohost.com.ezproxy.library.uq.edu.au/ehost/viewarticle?data=dGJyMPPp44rp2%2FdV0%2Bnjisfk5Ie46a9It62zT6%2Bk63nn5Kx95uXxjL6urVGtqK5JsJa1UrCquE2vls5lpOrweezp33vy3%2B2G59q7SbKqr1CzqLVOspzqeezdu33snOJ6u9nnhrCmpIzf3btZzJzfhruorkmuqrZLs6q0PuTl8IXf6rt%2B8%2BLqjOPu8gAA&hid=113) | USA | Clinical Ax  Imaging referral  Serology referral  Acute CN Mx |
| 26 | Wukich DK, Sung W. (2009) | [Charcot arthropathy of the foot and ankle: modern concepts and management review](https://ovidsp-tx-ovid-com.cknservices.dotsec.com/sp-3.5.1a/ovidweb.cgi?&S=NCAIFPLOLCDDIODMNCPKFEJCKNFCAA00&Complete+Reference=S.sh.37|82|1) | Netherlands | Clinical Signs/Symptoms  Clinic Ax  Imaging referral  Acute CN Mx  Surgical referral |
| 27 | Bernstein B, Motko J. (2008) | [Developing a comprehensive diagnostic and treatment plan for charcot Neuroarthropathy--Pt. 1](http://web.ebscohost.com.ezproxy.library.uq.edu.au/ehost/viewarticle?data=dGJyMPPp44rp2%2FdV0%2Bnjisfk5Ie46a9It62zT6%2Bk63nn5Kx95uXxjL6urVGtqK5JsJa0UrKvuEmwls5lpOrweezp33vy3%2B2G59q7SbKqr1CzqLVOspzqeezdu33snOJ6u9nnhrCmpIzf3btZzJzfhruorki3r7JItKe0PuTl8IXf6rt%2B8%2BLqjOPu8gAA&hid=113) | USA | Clinical Signs/Symptoms  Serology referrals  Acute CN Mx  LT Mx |
| 28 | Bernstein B, Motko J. (2008) | [Developing a comprehensive diagnostic and treatment plan for Charcot neuroarthropathy -- part 2](http://web.ebscohost.com.ezproxy.library.uq.edu.au/ehost/viewarticle?data=dGJyMPPp44rp2%2FdV0%2Bnjisfk5Ie46a9It62zT6%2Bk63nn5Kx95uXxjL6urVGtqK5JsJa0UrKvuEmwls5lpOrweezp33vy3%2B2G59q7SbKqr1CzqLVOspzqeezdu33snOJ6u9nnhrCmpIzf3btZzJzfhruorki3rrRQta6yPuTl8IXf6rt%2B8%2BLqjOPu8gAA&hid=113) | USA | Surgical referral |
| 29 | Crews RT, Wrobel JS. (2008) | [Physical management of the Charcot foot](https://ovidsp-tx-ovid-com.cknservices.dotsec.com/sp-3.5.1a/ovidweb.cgi?&S=NCAIFPLOLCDDIODMNCPKFEJCKNFCAA00&Complete+Reference=S.sh.37|151|1) | UK | Acute CN Mx  LT Mx |
| 30 | Dattani R, Patnaik S, Lal M. (2008) | [Charcot foot](https://ovidsp-tx-ovid-com.cknservices.dotsec.com/sp-3.5.1a/ovidweb.cgi?&S=NCAIFPLOLCDDIODMNCPKFEJCKNFCAA00&Complete+Reference=S.sh.37|130|1) | UK | Acute CN Mx |
| 31 | Jeffcoate WJ. (2008) | [Charcot neuro-osteoarthropathy](https://ovidsp-tx-ovid-com.cknservices.dotsec.com/sp-3.5.1a/ovidweb.cgi?&S=NCAIFPLOLCDDIODMNCPKFEJCKNFCAA00&Complete+Reference=S.sh.37|136|1) | USA | Clinical Ax |
| 32 | Jostel A, Jude EB. (2008) | [Medical treatment of Charcot neuroosteoarthropathy](https://ovidsp-tx-ovid-com.cknservices.dotsec.com/sp-3.5.1a/ovidweb.cgi?&S=NCAIFPLOLCDDIODMNCPKFEJCKNFCAA00&Complete+Reference=S.sh.37|152|1) | UK | Clinical Signs/Symptoms  Ddx  Acute CN Mx |
| 33 | Naqvi A, Cuchacovich R, Saketkoo L, Espinoza LR. (2008) | [Acute Charcot arthropathy successfully treated with pamidronate: long-term follow-up](https://ovidsp-tx-ovid-com.cknservices.dotsec.com/sp-3.5.1a/ovidweb.cgi?&S=NCAIFPLOLCDDIODMNCPKFEJCKNFCAA00&Link+Set=S.sh.37|146|sl_10) | USA | Serology referral  Acute CN Mx |
| 34 | Ndip A, Jude EB, Whitehouse R, Prescott M, Boulton AJ. (2008) | [Charcot neuroarthropathy triggered by osteomyelitis and/or surgery](https://ovidsp-tx-ovid-com.cknservices.dotsec.com/sp-3.5.1a/ovidweb.cgi?&S=NCAIFPLOLCDDIODMNCPKFEJCKNFCAA00&Link+Set=S.sh.37|119|sl_10) | UK | Clinical Ax  Imaging referral |
| 35 | Nielson DL, Armstrong DG. (2008) | The natural history of Charcot's neuroarthropathy | UK | Acute CN Mx |
| 36 | Petrova NL, Edmonds ME. (2008) | [Charcot neuro-osteoarthropathy-current standards](https://ovidsp-tx-ovid-com.cknservices.dotsec.com/sp-3.5.1a/ovidweb.cgi?&S=NCAIFPLOLCDDIODMNCPKFEJCKNFCAA00&Complete+Reference=S.sh.37|135|1) | USA | Clinical Ax  Imaging referral  Serology referral  Ddx  Chronic CN Dx criteria  LT Mx |
| 37 | Rogers LC, Bevilacqua NJ. (2008) | [The diagnosis of Charcot foot](https://ovidsp-tx-ovid-com.cknservices.dotsec.com/sp-3.5.1a/ovidweb.cgi?&S=NCAIFPLOLCDDIODMNCPKFEJCKNFCAA00&Complete+Reference=S.sh.37|153|1) | UK | Clinical Signs/Symptoms  Clinical Ax  Imaging referral |
| 38 | Rogers LC, Bevilacqua NJ. (2008) | [Imaging of the Charcot foot](https://ovidsp-tx-ovid-com.cknservices.dotsec.com/sp-3.5.1a/ovidweb.cgi?&S=NCAIFPLOLCDDIODMNCPKFEJCKNFCAA00&Complete+Reference=S.sh.37|142|1) | UK | Imaging referral |
| 39 | Stanley JC, Collier AM. (2008) | [Charcot osteo-arthropathy](http://web.ebscohost.com.ezproxy.library.uq.edu.au/ehost/viewarticle?data=dGJyMPPp44rp2%2FdV0%2Bnjisfk5Ie46a9It62zT6%2Bk63nn5Kx95uXxjL6urVGtqK5JsJa1UrCquE2vls5lpOrweezp33vy3%2B2G59q7SbKqr1CzqLVOspzqeezdu33snOJ6u9nnhrCmpIzf3btZzJzfhruorkmup7NOr6quPuTl8IXf6rt%2B8%2BLqjOPu8gAA&hid=113) | USA | Clinical Ax  Imaging referral |
| 40 | Ulbrecht JS, Wukich DK. (2008) | [The Charcot foot: medical and surgical therapy](https://ovidsp-tx-ovid-com.cknservices.dotsec.com/sp-3.5.1a/ovidweb.cgi?&S=NCAIFPLOLCDDIODMNCPKFEJCKNFCAA00&Complete+Reference=S.sh.37|121|1) | USA | Clinical Ax  Imaging referral  Acute CN Mx  LT Mx |
| 41 | Vella S, Cachia MJ. (2008) | [Charcot neuroarthropathy: Pathogenesis diagnosis and medical management](https://ovidsp-tx-ovid-com.cknservices.dotsec.com/sp-3.5.1a/ovidweb.cgi?&S=KHIGFPGMKJDDIOMENCPKFAOBDLLOAA00&Complete+Reference=S.sh.42|37|1) | Morocco | Clinical Ax  Imaging referral Acute CN Mx  Chronic CN Dx criteria |
| 42 | Wukich DK, Belczyk RJ. (2008) | [Foot & Ankle Special Interest Group. Stage 0 Charcot](http://web.ebscohost.com.ezproxy.library.uq.edu.au/ehost/viewarticle?data=dGJyMPPp44rp2%2FdV0%2Bnjisfk5Ie46a9It62zT6%2Bk63nn5Kx95uXxjL6urVGtqK5JsJa1UrCquE2vls5lpOrweezp33vy3%2B2G59q7SbKqr1CzqLVOspzqeezdu33snOJ6u9nnhrCmpIzf3btZzJzfhruorkmupq5LrqqwPuTl8IXf6rt%2B8%2BLqjOPu8gAA&hid=113) | USA | Acute CN Mx |
| 43 | Chantelau E, Kimmerle R, Poll LW. (2007) | [Nonoperative treatment of neuro-osteoarthropathy of the foot: do we need new criteria?](https://ovidsp-tx-ovid-com.cknservices.dotsec.com/sp-3.5.1a/ovidweb.cgi?&S=NCAIFPLOLCDDIODMNCPKFEJCKNFCAA00&Complete+Reference=S.sh.37|171|1) | UK | Clinical Ax  Acute CN Mx  LT Mx |
| 44 | Choksi P, Thomas R, Simmons DL. (2007) | [Charcot arthropathy. An often overlooked complication of diabetes mellitus](https://ovidsp-tx-ovid-com.cknservices.dotsec.com/sp-3.5.1a/ovidweb.cgi?&S=NCAIFPLOLCDDIODMNCPKFEJCKNFCAA00&Complete+Reference=S.sh.37|174|1) | Canada | Acute CN Mx |
| 45 | Harris TG, Harris TL Jr. (2007) | [Charcot neuroarthropathy: update and treatment options](http://web.ebscohost.com.ezproxy.library.uq.edu.au/ehost/viewarticle?data=dGJyMPPp44rp2%2FdV0%2Bnjisfk5Ie46a9It62zT6%2Bk63nn5Kx95uXxjL6urVGtqK5JsJa0UrKvuEmwls5lpOrweezp33vy3%2B2G59q7SbKqr1CzqLVOspzqeezdu33snOJ6u9nnhrCmpIzf3btZzJzfhruorki3q7JOs6uuPuTl8IXf6rt%2B8%2BLqjOPu8gAA&hid=113) | USA | Imaging referral  Acute CN Mx |
| 46 | Johnsen B. (2007) | [Acute Charcot's arthropathy: a difficult diagnosis](http://www.ncbi.nlm.nih.gov/pubmed/17695093) | USA | Clinical Ax  Imaging referral  Acute CN Mx |
| 47 | Matricali GA, Bammens B, Kuypers D, Flour M, Mathieu C. (2007) | [High rate of Charcot foot attacks early after simultaneous pancreas-kidney transplantation](https://ovidsp-tx-ovid-com.cknservices.dotsec.com/sp-3.5.1a/ovidweb.cgi?&S=NCAIFPLOLCDDIODMNCPKFEJCKNFCAA00&Link+Set=S.sh.37|177|sl_10) | USA | Serology referral - renal |
| 48 | Ore HM, Watkinson D. (2007) | [Case study. Charcot's neuropathy](http://web.ebscohost.com.ezproxy.library.uq.edu.au/ehost/viewarticle?data=dGJyMPPp44rp2%2FdV0%2Bnjisfk5Ie46a9It62zT6%2Bk63nn5Kx95uXxjL6urVGtqK5JsJa2Uq6ruEqxls5lpOrweezp33vy3%2B2G59q7SbKqr1CzqLVOspzqeezdu33snOJ6u9nnhrCmpIzf3btZzJzfhruorki3rLBKsqyyPuTl8IXf6rt%2B8%2BLqjOPu8gAA&hid=113) | UK | Serology referral  Acute CN Mx |
| 49 | Pinzur MS. (2007) | [Current concepts review: Charcot arthropathy of the foot and ankle](https://ovidsp-tx-ovid-com.cknservices.dotsec.com/sp-3.5.1a/ovidweb.cgi?&S=NCAIFPLOLCDDIODMNCPKFEJCKNFCAA00&Complete+Reference=S.sh.37|165|1) | USA | Clinical Ax  Imaging referral  Acute CN Mx |
| 50 | [Tan PL](http://www.ncbi.nlm.nih.gov/pubmed?term=Tan PL%5BAuthor%5D&cauthor=true&cauthor_uid=16687463), [Teh J](http://www.ncbi.nlm.nih.gov/pubmed?term=Teh J%5BAuthor%5D&cauthor=true&cauthor_uid=16687463). (2007) | MRI of the diabetic foot: differentiation of infection from neuropathic change | UK | Imaging referral |
| 51 | Soysal N, Ayhan M, Guney E, Akyol A. (2007) | [Differential diagnosis of Charcot arthropathy and osteomyelitis](https://ovidsp-tx-ovid-com.cknservices.dotsec.com/sp-3.5.1a/ovidweb.cgi?&S=NCAIFPLOLCDDIODMNCPKFEJCKNFCAA00&Complete+Reference=S.sh.37|158|1) | Sweden | Serology referral  Imaging referral |
| 52 | Zgonis T, Stapleton JJ, Shibuya N, Roukis TS. (2007) | [Surgically induced Charcot neuroarthropathy following partial forefoot amputation in diabetes](https://ovidsp-tx-ovid-com.cknservices.dotsec.com/sp-3.5.1a/ovidweb.cgi?&S=NCAIFPLOLCDDIODMNCPKFEJCKNFCAA00&Complete+Reference=S.sh.37|175|1) | UK | Clinical Ax |
| 53 | Andersen LB, Dipreta J. (2006) | [Charcot of the calcaneus](https://ovidsp-tx-ovid-com.cknservices.dotsec.com/sp-3.5.1a/ovidweb.cgi?&S=NCAIFPLOLCDDIODMNCPKFEJCKNFCAA00&Complete+Reference=S.sh.37|181|1) | UK | Imaging referral  Acute CN Mx |
| 54 | Buttke J. (2006) | [Identifying the Charcot foot](https://ovidsp-tx-ovid-com.cknservices.dotsec.com/sp-3.5.1a/ovidweb.cgi?&S=NCAIFPLOLCDDIODMNCPKFEJCKNFCAA00&Complete+Reference=S.sh.37|195|1) | USA | Clinical Signs/Symptoms  Clinical Ax  Serology referral  Ddx |
| 55 | Chantelau E, Richter A, Schmidt-Grigoriadis P, Scherbaum WA. (2006) | [The diabetic charcot foot: MRI discloses bone stress injury as trigger mechanism of neuroarthropathy](https://ovidsp-tx-ovid-com.cknservices.dotsec.com/sp-3.5.1a/ovidweb.cgi?&S=KHIGFPGMKJDDIOMENCPKFAOBDLLOAA00&Complete+Reference=S.sh.42|64|1) | Germany | Imaging referral |
| 56 | Frykberg RG. Zgonis T. Armstrong DG. Driver VR. Giurini JM. Kravitz SR. Landsman AS. Lavery LA. Moore JC. Schuberth JM. Wukich DK. Andersen C. Vanore JV. American College of Foot and Ankle Surgeons. (2006) | [Diabetic foot disorders. A clinical practice guideline (2006 revision)](https://ovidsp-tx-ovid-com.cknservices.dotsec.com/sp-3.5.1a/ovidweb.cgi?&S=NCAIFPLOLCDDIODMNCPKFEJCKNFCAA00&Complete+Reference=S.sh.37|178|1) | UK | Imaging referral  Serology referral  Imaging referral  Acute CN Mx  LT Mx |
| 57 | Giurato L, Uccioli L. (2006) | [The diabetic foot: Charcot joint and osteomyelitis](https://ovidsp-tx-ovid-com.cknservices.dotsec.com/sp-3.5.1a/ovidweb.cgi?&S=NCAIFPLOLCDDIODMNCPKFEJCKNFCAA00&Link+Set=S.sh.37|187|sl_10) | USA | Clinical Ax  Imaging referral  Serology referral |
| 58 | Hunt A. (2006) | [The treatment of Charcot neuroarthropathy with an Aircast PneumaticWalker: a case study](http://web.ebscohost.com.ezproxy.library.uq.edu.au/ehost/viewarticle?data=dGJyMPPp44rp2%2FdV0%2Bnjisfk5Ie46a9It62zT6%2Bk63nn5Kx95uXxjL6urVGtqK5JsJa0UrKvuEmwls5lpOrweezp33vy3%2B2G59q7SbKqr1CzqLVOspzqeezdu33snOJ6u9nnhrCmpIzf3btZzJzfhruorki3qLFIt660PuTl8IXf6rt%2B8%2BLqjOPu8gAA&hid=113) | UK | Acute CN Mx |
| 59 | Salgami EV, Bowling FL, Whitehouse RW, Boulton AJ. (2006) | [Charcot neuroarthropathy: an unusual case and a review of the literature](https://ovidsp-tx-ovid-com.cknservices.dotsec.com/sp-3.5.1a/ovidweb.cgi?&S=NCAIFPLOLCDDIODMNCPKFEJCKNFCAA00&Complete+Reference=S.sh.37|185|1) | USA | Introduction  Clinical Ax  Imaging referral  Acute CN Mx |
| 60 | Buttke J. (2005) | [Stepping up foot injury diagnosis: Jones, Lisfranc, and Charcot](https://ovidsp-tx-ovid-com.cknservices.dotsec.com/sp-3.5.1a/ovidweb.cgi?&S=NCAIFPLOLCDDIODMNCPKFEJCKNFCAA00&Link+Set=S.sh.37|199|sl_10) | USA | Ddx  Serology referral  Imaging referral |
| 61 | Hastings MK, Sinacore DR, Fielder FA, Johnson JE. (2005) | [Bone mineral density during total contact cast immobilization for a patient with neuropathic (Charcot) arthropathy](https://ovidsp-tx-ovid-com.cknservices.dotsec.com/sp-3.5.1a/ovidweb.cgi?&S=PFPJFPOMAEDDIOLENCPKKHFBFFDLAA00&Complete+Reference=S.sh.37|215|1) | USA | Acute CN MX  Clinical Ax  LT Mx |
| 62 | Ledermann HP, Morrison WB. (2005) | [Differential diagnosis of pedal osteomyelitis and diabetic neuroarthropathy: MR imaging](https://ovidsp-tx-ovid-com.cknservices.dotsec.com/sp-3.5.1a/ovidweb.cgi?&S=KHIGFPGMKJDDIOMENCPKFAOBDLLOAA00&Complete+Reference=S.sh.42|56|1) | USA | Imaging referral  Ddx |
| 63 | McConville DO, Archbold P, McKeown D. (2005) | [Management of a charcot joint. Considerations in the use of an AIRCAST boot](http://web.ebscohost.com.ezproxy.library.uq.edu.au/ehost/viewarticle?data=dGJyMPPp44rp2%2FdV0%2Bnjisfk5Ie46a9It62zT6%2Bk63nn5Kx95uXxjL6urVGtqK5JsJa2Uq6ruEqxls5lpOrweezp33vy3%2B2G59q7SbKqr1CzqLVOspzqeezdu33snOJ6u9nnhrCmpIzf3btZzJzfhruorkizprZOsKm0PuTl8IXf6rt%2B8%2BLqjOPu8gAA&hid=113) | UK | Clinical Ax  Acute CN Mx  LT Mx |
| 64 | Trepman E, Nihal A, Pinzur MS. (2005) | [Current topics review: Charcot neuroarthropathy of the foot and ankle](https://ovidsp-tx-ovid-com.cknservices.dotsec.com/sp-3.5.1a/ovidweb.cgi?&S=PFPJFPOMAEDDIOLENCPKKHFBFFDLAA00&Complete+Reference=S.sh.37|217|1) | USA | Clinical Signs/Symptoms  Clinical Ax  Ddx  Imaging referral  Acute CN Mx  LT Mx |
| 65 | Berendt AR, Lipsky B. (2004) | [Is this bone infected or not? Differentiating neuro-osteoarthropathy from osteomyelitis in the diabetic foot](https://ovidsp-tx-ovid-com.cknservices.dotsec.com/sp-3.5.1a/ovidweb.cgi?&S=KHIGFPGMKJDDIOMENCPKFAOBDLLOAA00&Complete+Reference=S.sh.42|79|1) | USA | Imaging referral  Ddx – infection/OM  Serology referral |
| 66 | Gill GV, Hayat H, Majid S. (2004) | [Diagnostic delays in diabetic Charcot arthropathy](https://ovidsp-tx-ovid-com.cknservices.dotsec.com/sp-3.5.1a/ovidweb.cgi?&S=NBDAFPIKLFDDIOFONCPKKHIBKLCDAA00&Complete+Reference=S.sh.38|280|1) | USA | Clinical Signs/Symptoms  Clinical Ax  Acute CN Mx  Ddx |
| 67 | Slater RA, Ramot Y, Buchs A, Rapoport MJ. (2004) | [The diabetic Charcot foot](https://ovidsp-tx-ovid-com.cknservices.dotsec.com/sp-3.5.1a/ovidweb.cgi?&S=PFPJFPOMAEDDIOLENCPKKHFBFFDLAA00&Complete+Reference=S.sh.37|229|1) | Israel | Clinical Signs/Symptoms  Clinical Ax  Ddx  Imaging referral  Acute CN Mx  LT Mx  Chronic CN Dx Criteria |
| 68 | Amital H, Applbaum YH, Bar-On H, Rubinow A. (2003) | [Inside a Charcot joint](https://ovidsp-tx-ovid-com.cknservices.dotsec.com/sp-3.5.1a/ovidweb.cgi?&S=PFPJFPOMAEDDIOLENCPKKHFBFFDLAA00&Complete+Reference=S.sh.37|241|1) | Israel | Clinical Ax  Imaging referral |
| 69 | Graves M, Tarquinio TA. (2003) | [Diabetic neuroarthropathy (Charcot joints): the importance of recognizing chronic sensory deficits in the treatment of acute foot and ankle fractures in diabetic patients](https://ovidsp-tx-ovid-com.cknservices.dotsec.com/sp-3.5.1a/ovidweb.cgi?&S=PFPJFPOMAEDDIOLENCPKKHFBFFDLAA00&Complete+Reference=S.sh.37|243|1) | USA | Clinical Signs/Symptoms  Clinical Ax  Ddx  Serology referral  Imaging referral  Acute CN Mx |
| 70 | Gupta PP, Mohan V. (2003) | [Charcot foot--an update](https://ovidsp-tx-ovid-com.cknservices.dotsec.com/sp-3.5.1a/ovidweb.cgi?&S=PFPJFPOMAEDDIOLENCPKKHFBFFDLAA00&Complete+Reference=S.sh.37|242|1) | India | Clinical Signs/Symptoms  Clinical Ax  Ddx  Imaging referral  Acute CN Mx |
| 71 | Juliano PJ, Harris TG. (2003) | [Charcot foot: Update, diagnosis, treatment, reconstruction, and limb salvage](https://ovidsp-tx-ovid-com.cknservices.dotsec.com/sp-3.5.1a/ovidweb.cgi?&S=KHIGFPGMKJDDIOMENCPKFAOBDLLOAA00&Link+Set=S.sh.42|86|sl_10) | USA | Ddx  Serology referral  Imaging referral  Chronic CN dx criteria  Acute CN Mx |
| 72 | Lee L, Blume PA, Sumpio B. (2003) | [Charcot joint disease in diabetes mellitus](https://ovidsp-tx-ovid-com.cknservices.dotsec.com/sp-3.5.1a/ovidweb.cgi?&S=PFPJFPOMAEDDIOLENCPKKHFBFFDLAA00&Complete+Reference=S.sh.37|234|1) | Germany | Clinical Signs/Symptoms  Clinical Ax  Ddx  Imaging referral  Acute CN Mx |
| 73 | Saleem TFM, Caputo GM, Juliano PJ, Ulbrecht JS. (2003) | [Recognizing and managing Charcot foot](http://web.ebscohost.com.ezproxy.library.uq.edu.au/ehost/viewarticle?data=dGJyMPPp44rp2%2FdV0%2Bnjisfk5Ie46a9It62zT6%2Bk63nn5Kx95uXxjL6urVGtqK5JsJa1UrCquE2vls5lpOrweezp33vy3%2B2G59q7SbKqr1CzqLVOspzqeezdu33snOJ6u9nnhrCmpIzf3btZzJzfhruorkiyprRJta60PuTl8IXf6rt%2B8%2BLqjOPu8gAA&hid=113) | UK | Clinical Signs/Symptoms  Clinical Ax  Ddx  Acute CN Mx  LT Mx |
| 74 | [Armstrong DG](http://www.ncbi.nlm.nih.gov/pubmed?term=Armstrong DG%5BAuthor%5D&cauthor=true&cauthor_uid=12122125), [Peters EJ](http://www.ncbi.nlm.nih.gov/pubmed?term=Peters EJ%5BAuthor%5D&cauthor=true&cauthor_uid=12122125). (2002) | [Charcot's Arthropathy of the foot](http://www.ncbi.nlm.nih.gov/pubmed/12122125) | USA | Introduction  Clinical Signs/Symptoms  Clinical Ax  Imaging referral  Acute CN Mx  Ddx  Criteria for Chronic Dx |
| 75 | Hartemann-Heurtier A, Van GH, Grimaldi A. (2002) | [The Charcot foot](https://ovidsp-tx-ovid-com.cknservices.dotsec.com/sp-3.5.1a/ovidweb.cgi?&S=PFPJFPOMAEDDIOLENCPKKHFBFFDLAA00&Complete+Reference=S.sh.37|251|1) | UK | Ddx  Serology referral  Clinical Signs/Symptoms  Clinical Ax  Acute CN Mx  Criteria for Chronic Dx  LT Mx  Surgical referral |
| 76 | Jude EB, Boulton AJ. (2002) | [Medical treatment of Charcot's Arthropathy](http://www.ncbi.nlm.nih.gov/pubmed/12122123) | USA | Acute CN Mx |
| 77 | [Nubé VL](http://www.ncbi.nlm.nih.gov/pubmed?term=Nubé VL%5BAuthor%5D&cauthor=true&cauthor_uid=12122124), [McGill M](http://www.ncbi.nlm.nih.gov/pubmed?term=McGill M%5BAuthor%5D&cauthor=true&cauthor_uid=12122124), [Molyneaux L](http://www.ncbi.nlm.nih.gov/pubmed?term=Molyneaux L%5BAuthor%5D&cauthor=true&cauthor_uid=12122124), [Yue DK](http://www.ncbi.nlm.nih.gov/pubmed?term=Yue DK%5BAuthor%5D&cauthor=true&cauthor_uid=12122124). (2002) | From Acute to Chronic: Monitoring the Progress of Charcot’s Arthropathy | USA | Clinical Signs/Symptoms  Clinical Ax  Imaging referral  Acute CN Mx  LT Mx  Surgical referral |
| 78 | Rajbhandari SM, Jenkins RC, Davies C, Tesfaye S. (2002) | [Charcot neuroarthropathy in diabetes mellitus](https://ovidsp-tx-ovid-com.cknservices.dotsec.com/sp-3.5.1a/ovidweb.cgi?&S=PFPJFPOMAEDDIOLENCPKKHFBFFDLAA00&Complete+Reference=S.sh.37|255|1) | Europe | Clinical Signs/Symptoms  Clinical Ax  Ddx  Imaging referral  Acute CN Mx  LT Mx |
| 79 | Yu GV, Hudson JR. (2002) | [Evaluation and treatment of stage 0 Charcot's neuroarthropathy of the foot and ankle](https://ovidsp-tx-ovid-com.cknservices.dotsec.com/sp-3.5.1a/ovidweb.cgi?&S=PFPJFPOMAEDDIOLENCPKKHFBFFDLAA00&Complete+Reference=S.sh.37|260|1) | USA | Clinical Signs/Symptoms  Clinical Ax  Serology referral  Imaging referral  Acute CN Mx  LT Mx |
|  |  | | | |
